# A systematic review of therapeutic hypothermia for adult patients following traumatic brain injury

**DOI:** 10.1186/cc13835

**Published:** 2014-04-17

**Authors:** Samantha Crossley, Jenny Reid, Rachel McLatchie, Judith Hayton, Clair Clark, Margaret MacDougall, Peter JD Andrews

**Affiliations:** 1 Medical Researcher, University of Edinburgh, Edinburgh, UK; 2 Medical Statistician and Researcher at the University of Edinburgh, Edinburgh, UK; 3 Anesthetics & Intensive Care at the University of Edinburgh and Consultant in Anesthesia and Intensive Care at the Western General Hospital, Lothian University Hospitals Division, Edinburgh EH4 2XU, UK

## Abstract

**Introduction:**

Research into therapeutic hypothermia following traumatic brain injury has been characterised by small trials of poor methodological quality, producing variable results. The Cochrane review, published in 2009, now requires updating. The aim of this systematic review is to assess the effectiveness of the application of therapeutic hypothermia to reduce death and disability when administered to adult patients who have been admitted to hospital following traumatic brain injury.

**Methods:**

Two authors extracted data from each trial. Unless stated in the trial report, relative risks and 95% confidence intervals (CIs) were calculated for each trial. We considered *P* < 0 · 05 to be statistically significant. We combined data from all trials to estimate the pooled risk ratio (RR) with 95% confidence intervals for death, unfavourable outcome, and pneumonia. All statistical analyses were performed using RevMan 5.1 (Cochrane IMS, Oxford, UK) and Stata (Intercooled Version 12.0, StataCorp LP). Pooled RRs were calculated using the Mantel-Haenszel estimator. The random effects model of DerSimonian and Laird was used to estimate variances for the Mantel-Haenszel and inverse variance estimators.

**Results:**

Twenty studies are included in the review, while 18 provided mortality data. When the results of 18 trials that evaluated mortality as one of the outcomes were statistically aggregated, therapeutic hypothermia was associated with a significant reduction in mortality and a significant reduction in poor outcome. There was a lack of statistical evidence for an association between use of therapeutic hypothermia and increased onset of new pneumonia.

**Conclusions:**

In contrast to previous reviews, this systematic review found some evidence to suggest that therapeutic hypothermia may be of benefit in the treatment of traumatic brain injury. The majority of trials were of low quality, with unclear allocation concealment. Low quality trials may overestimate the effectiveness of hypothermia treatment versus standard care. There remains a need for more, high quality, randomised control trials of therapeutic hypothermia after traumatic brain injury.

PROSPERO Systematic Review Registration Number 2012: CRD42012002449.

## Introduction

Therapeutic hypothermia has emerged as a potentially life-saving treatment for the care of the critically ill. Research in the 1980s using animal models demonstrated the benefits of cooling to 32 to 34°C [1,2], and it has since been proposed that there are a number of potential applications for therapeutic hypothermia [3]. In February 2011, National Health Service (NHS) National Institute for Health and Clinical Excellence (NICE) guidelines were published to support the use of therapeutic hypothermia for hypoxic ischaemic encephalopathy [4]. Similarly, NICE guidelines for the use of therapeutic hypothermia in cardiac arrest have also been published [5]. In the United States, the American Heart Association recommends hypothermia as a standard of care for survivors of cardiac arrest as there is sufficient evidence to support improvements in outcome with its use [6]. Whilst a number of studies have identified an improvement in outcome with the application of therapeutic hypothermia following stroke [7], the question as to whether therapeutic hypothermia is of benefit in traumatic brain injury (TBI) remains unanswered.

TBI is a leading cause of disability and death, particularly in the young [8]. The application of hypothermia has been shown to decrease cerebral metabolic rate and is thought to alter the release of post-trauma excitatory neurotransmitters [9], reducing and preventing blood-brain-barrier disruptions during and following cerebral insults [10]. Laboratory studies have shown positive benefits for prophylactic hypothermia, but clinical trials and systematic reviews have largely suggested weak evidence to support the use of therapeutic hypothermia following TBI [10-16]. Brain Trauma Foundation Guidelines are most widely followed and do not recommend the use of therapeutic hypothermia (level III evidence) [11] and there have also been concerns about possible increased risk of pneumonia following the induction of therapeutic hypothermia [9].

A number of reviews have been published into the use of therapeutic hypothermia following TBI. This systematic review was designed to identify all randomised controlled trials (RCTs) that investigate the relationship between TBI and the application of therapeutic hypothermia in adults.

### Aims

Our primary aim was to assess the effect of the application of therapeutic hypothermia, when administered to adult patients who have been admitted to intensive care following TBI, on the risk of death, unfavourable outcome, and new pneumonia.

Our secondary aims were to investigate the following hypotheses: a) duration of cooling greater than 48 hours improves outcome compared with shorter duration; b) re-warming patients at a speed of greater than 1°C every four hours increases the risk of poor outcome; c) patients who have undergone only modest cooling (35 to 36°C) are more likely to experience poor outcomes compared with patients cooled to below 35°C; and d) increased length of time between the onset of injury and the induction of cooling increases the risk of poor outcome.

## Materials and methods

### Search methods for identification of trials

Searches were not restricted by date, language, or publication status (other than those restrictions imposed by the databases themselves). Cochrane Central Register of Controlled Trials (CENTRAL), MEDLINE, EMBASE, ISI Web of Science: Science Citation Index Expanded (SCI-EXPANDED) and Conference Proceedings Citation Index-Science (CPCI-S), PubMed, and Zetoc were searched from the first publicly accessible date of a particular database to 5 January 2012. All electronic database searches were conducted using versions of the MEDLINE search strategy, adapted where necessary for each database. The search strategy is recorded in the protocol (see Additional file 1).

### Other sources

Reference lists of all relevant trials and review articles were also hand searched and where necessary, authors were contacted to find information relevant to trials or conference proceedings.

### Ethical approval and consent

This study did not need ethical approval nor was individual patient consent needed.

### Selection of papers

#### Methodological criteria

The inclusion criterion was that trials must be RCTs. The exclusion criteria were trials in which patients had not been randomised to each treatment arm and/or where there was no control group managed to normothermia.

The condition or domain being studied was the application of therapeutic hypothermia for the treatment of TBI. We defined TBI as being any acute closed head injury sustained following head trauma. We defined therapeutic hypothermia as any intervention carried out with the intention of reducing core body temperature to below the physiological norm (36.0°C). Unfavourable outcomes at the end of the follow-up period included death, persistent vegetative state or severe disability as defined by the Glasgow outcome scale (GOS) or equivalent scoring scale (Ranchos Los Amigos scale).

### Participants/population

The inclusion criteria were that patients must be adults (we defined this as being the legal age for consent in the country in which the trial was conducted) and patients enrolled must have had closed head injuries. The exclusion criteria were trials that had been performed entirely in neonates or children (whom we define as being below the legal age for consent) and trials containing patients with open head injuries, such as gunshot wounds.

The output of the searches was exported into Endnote Web and five individuals sifted the papers in three separate phases: in sift phase one, the primary output of the searches was assessed. Papers with titles and abstracts unrelated to therapeutic hypothermia as a medical intervention or to the general management of TBI were discarded. This was recorded in Endnote web. In sift phase one, no reason was recorded for exclusion. This was deemed appropriate because all excluded papers had no relevance to therapeutic hypothermia or TBI.

In sift phase two, the abstracts of all remaining papers were assessed against the inclusion and exclusion criteria. If excluded, a reason was recorded. Reasons for exclusion in this phase included, but were not limited to: neonatal or paediatric patients, non-human trials, reviews rather than clinical trials, and therapeutic hypothermia for reasons other than TBI such as cardiac arrest.

In sift phase three, each remaining paper was reviewed in full for final inclusion criteria. Reasons for exclusion were recorded and the eligibility of each paper for inclusion was assessed by at least two authors. Where authors were uncertain and discrepancies remained, a majority decision between the authors determined if the trial met inclusion criteria (papers excluded at sift phase three can be seen in Additional file 2). The search protocols and a PRISMA flow diagram (see Additional file 3) detailing the trial selection process are included in Additional files 1 and 3.

### Data extraction

Each trial was assessed by at least two individuals and the data extraction was recorded in a data extraction matrix. The following general information was extracted from each of the selected trials: trial name and date of publication, method of intervention, lowest body temperature obtained, duration of intervention, maximum time between initial injury and cooling, Glasgow coma scale on admission, neurological deterioration, speed of re-warming, whether a sample size calculation was performed and if it was adhered to, the number of patients, patient outcomes in the control and treatment groups, and the effect size.

In order to assess the methodological quality of each trial the authors recorded the allocation concealment and randomisation technique used, the blinding procedure, reporting of an intention-to-treat analysis, completeness of follow up, reasons for patient exclusion, and whether a protocol was published and supplied with the trial.

In addition, information was extracted on the following possible confounding factors: whether the control group was managed to normothermia (considered standard care), whether the control group was actively rewarmed on admission (associated with poor outcomes), whether the treatment arm received barbiturate treatment in addition to therapeutic hypothermia (associated with poor outcome), whether there were significant differences between the treatment and control sample populations, and whether the standard treatment was clearly outlined. These data were used to support domain-based assessment of the risk of bias for each trial (see Additional file 4).

### Bias

The methodology described for random sequence generation, allocation concealment, blinding of participants and personnel, blinding of outcome assessment, completeness of outcome data, and selective reporting were assessed in our data extraction process using the Cochrane Collaboration’s tool for assessing risk of bias [17].

There were a number of trials where randomisation was stated but the method was unclear (see Additional file 5). All selected trials were RCTs and met our inclusion criteria. Due to the nature of the intervention, blinding of trial personnel was not possible and is recognised as a continuing source of weakness amongst trials of therapeutic hypothermia.

The 20 included trials were assessed according to trial quality using domain-based assessment of risk of bias, including potential confounding factors (as described above) in the design. The assessments for each trial are included in Additional files 5 and 6. We recognize that Cochrane discourages the use of scoring systems when assessing trial quality, and our domain-based assessment of risk of bias output is simply presence or absence (0 or 1 and similar to Cochrane red or green) of the key domains used when assessing risk of bias. The total number of positive domains (maximum 15 domains) was used as a tool for an exploratory meta-regression analysis.

### Data synthesis

Review Manager (RevMan, Cochrane Collaboration, version 5.1) and Stata (Intercooled Version 12.0, StataCorp LP) were used for data sythesis.

### Statistical analyses

The relative risk and corresponding 95% CI for death, poor outcome, and pneumonia were extracted where they were available or were calculated where this was not stated in the original trial report. The overall relative risk, (RR_overall_) and corresponding 95% CIs were then evaluated using the Mantel-Haenszel approach, and the significance of RR_overall_ as an effect estimate was assessed in terms of the null hypothesis RR_overall_ = 1 using the *z*-test. Although the corresponding study hypotheses were one-sided, two-sided hypothesis tests were assumed, thus ensuring a more conservative approach to statistical significance.

Statistical evidence for heterogeneity between trials was assessed using the *Q*-test, and the *I*^2^ index was used as an estimate of the extent of between trial variability. As noted by Huedo-Medina *et al*. [18], when dealing with a small sample size, a non-significant result for the *Q*-test may result in erroneous selection of the fixed effects model. In order to mitigate this effect, where a fixed model was chosen on the basis of a lack of evidence for heterogeneity, corresponding results for a random effects analysis (not included here) were generated for comparative purposes.

### Analysis of evidence for bias in selection of trials or tendency of smaller trials to report stronger effects

Funnel plots and contour-enhanced funnel plots were used to assess bias, including publication bias, in choice of trials as well as a generic tendency for smaller trials to report stronger effects. It is possible that smaller trials, often single-centred, will have the largest treatment effect. In addition to surveying the above plots, the Begg and the Egger tests of small-study effects were used as a further means of testing for the above anomalies.

### Sensitivity analysis

Forest plot analyses were performed separately for all studies and for those at least risk of bias (assessed by using Cochrane domain-based evaluation). Exploratory meta-regression analyses were performed on the log-transformed domain-based evaluation sum-scores for each of the three principal treatment outcomes in order to test for an association between trial quality (risk of bias) and treatment effect (Additional file 7).

## Results

Included in the final selection were 20 trials. These trials enrolled 1,885 patients (see Additional file 6). All trials except two [19,20] recorded outcomes for death in the control and treatment groups at final follow-up. All trials reported the incidence of poor outcome (death, vegetative state, and long-term disability) in both the control and treatment groups.

Eight trials did not provide data on the occurrence of pneumonia during treatment in either the control or treatment groups [21-28]). For a summary of the characteristics of each trial please refer to Additional file 6.

**Figure 1:**
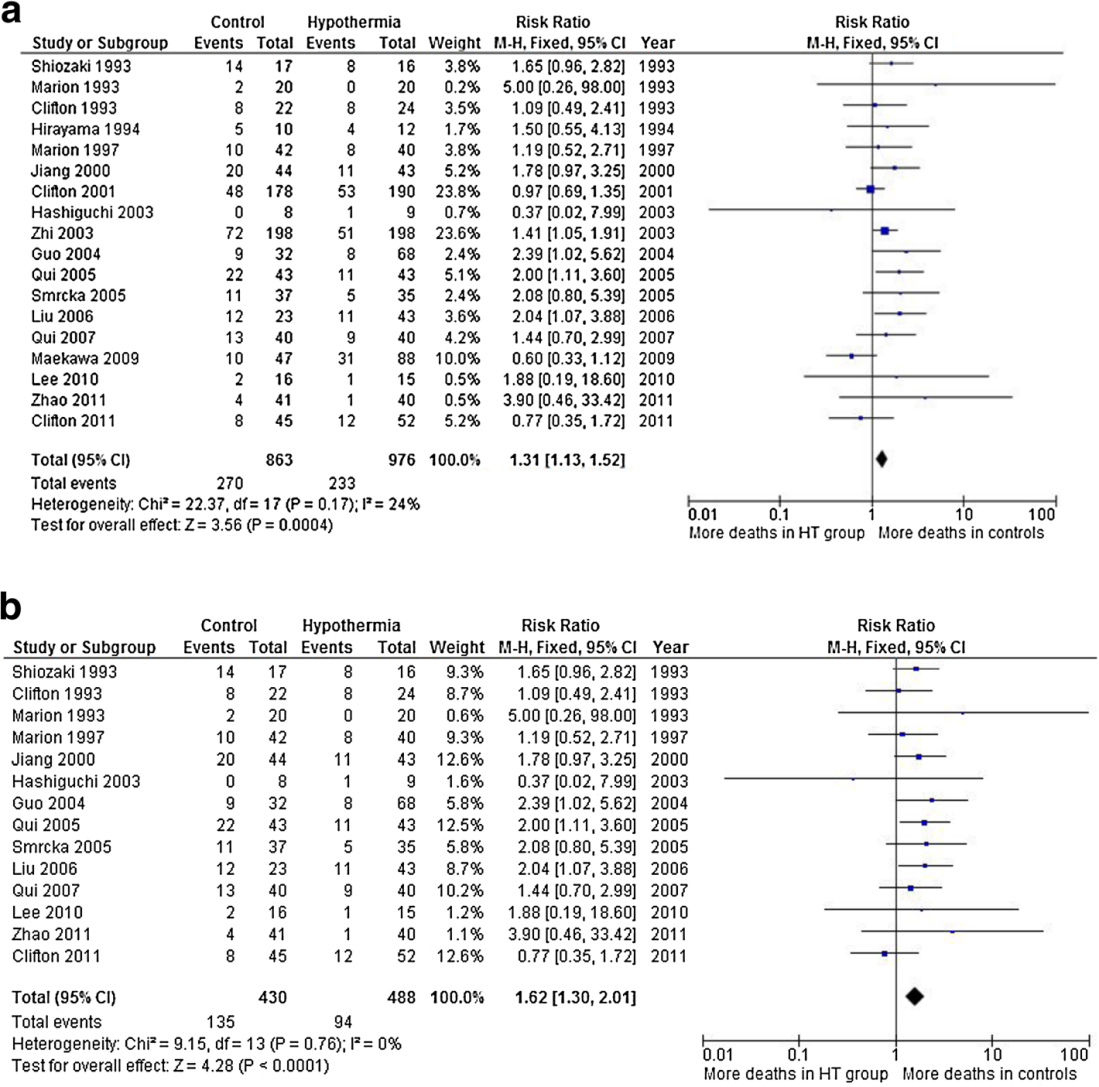
**Death at final follow-up. (a)** In total 18 trials involving 1,839 patients reported deaths. When the results of the 18 randomised controlled trials (RCTs) were statistically aggregated, therapeutic hypothermia was associated with a significant reduction in mortality (relative risk (RR) = 1.31, 95% CI = 1.13, 1.52, *P* = 0.0004). **(b)** Trials assessed as having lower risk of bias: when the results of the 14 RCTs were statistically aggregated, therapeutic hypothermia was associated with a significant reduction in mortality (RR = 1.62, 95% CI = 1.30, 2.01, *P* <0.0001).

In analysis of trials that reported death at final follow-up, 18 trials involving 1,839 patients reported deaths. When the results of the 18 RCTs were statistically aggregated, therapeutic hypothermia was associated with a significant reduction in mortality (relative risk (RR) = 1.31, 95% CI = 1.13 to 1.52, *P* = 0.0004). As the *Q*-test showed no evidence of statistical heterogeneity between the trials (χ^2^ = 22.37, *P* = 0.17, df = 17) a fixed-effects model was selected. This choice was supported by the low value of the *I*^2^ index (*I*^2^ = 24%). A random effects model was generated and the conclusions are unaffected Figure 1.

For death at final follow-up in trials assessed as lower risk of bias, 14 trials involving 918 patients and assessed as lower risk of bias (domain-based assessment) were included in this analysis. When the results of the 14 RCTs were statistically aggregated, therapeutic hypothermia was associated with a significant reduction in mortality (RR = 1.62, 95% CI = 1.30, 2.01, *P* <0.0001). As the *Q*-test showed a lack of evidence for statistical heterogeneity between the trials (χ^2^ = 9.15, *P* = 0.76, df = 13) a fixed effects model was selected. A random effects model was generated and the conclusions were unaffected (Figure 1b).

**Figure 2:**
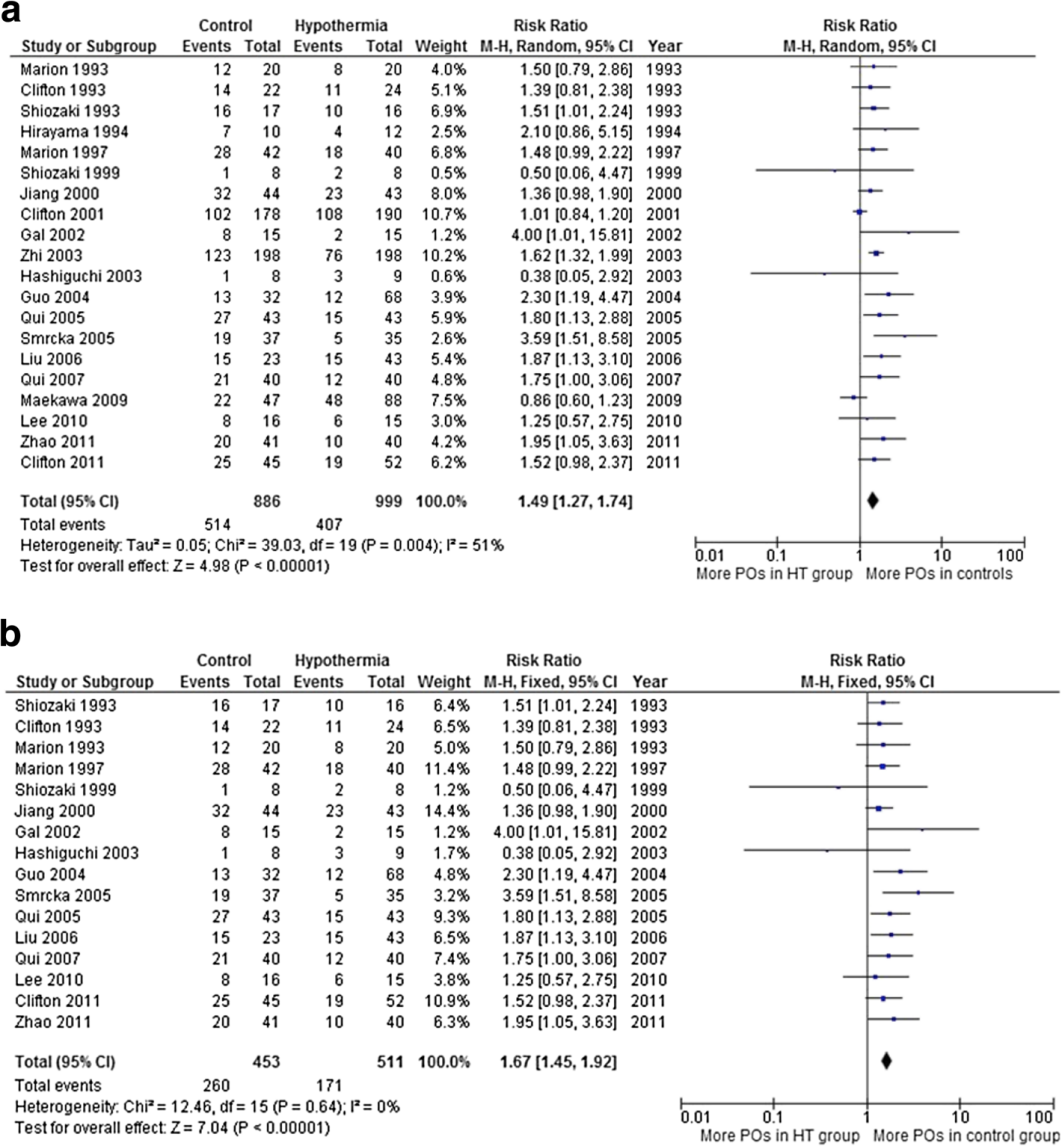
**Poor outcome at final follow-up. (a)** In total 20 trials involving 1,885 patients reported death, vegetative state, and long-term disability. When the results of 20 randomised controlled trials (RCTs) that evaluated poor outcome were statistically aggregated, therapeutic hypothermia was associated with a significant reduction in poor outcome (relative risk (RR) = 1.49, 95% CI = 1.27, 1.74, *P* <0.00001). **(b)** Trials assessed as lower risk of bias: 16 trials, involving 964 patients, and assessed as lower risk of bias (domain-based assessment) were included in this analysis. When the results of 16 RCTs that evaluated poor outcome were statistically aggregated, therapeutic hypothermia was associated with a significant reduction in poor outcome (RR = 1.67, 95% CI = 1.45, 1.92, *P* <0.00001).

In analysis of trials that reported poor outcome at final follow-up, 20 trials involving 1,885 patients reported death, vegetative state, and long-term disability. When the results of 20 RCTs that evaluated poor outcome were statistically aggregated, therapeutic hypothermia was associated with a significant reduction in poor outcome (RR = 1.49, 95% CI = 1.27, 1.74, *P* <0.00001). As the *Q*-test demonstrated that there was statistical evidence of heterogeneity between the trials (*P* = 0.004) [12], a random effects model was used. This choice was supported by the moderate value for the *I*^2^ index (*I*^2^ = 51%) (Figure 2).

For poor outcome at final follow-up in trials assessed as lower risk of bias, 16 trials, involving 964 patients, and assessed as lower risk of bias (domain-based assessment) were included in this analysis. When the results of 16 RCTs that evaluated poor outcome were statistically aggregated, therapeutic hypothermia was associated with a significant reduction in poor outcome (RR = 1.67, 95% CI = 1.45, 1.92, *P* <0.00001). As the *Q*-test showed a lack of statistical evidence for heterogeneity between the trials (χ^2^ = 12.46, *P* = 0.64, df = 15), a fixed effects model was selected. A random effects model was generated and the conclusions were unaffected (Figure 2b).

**Figure 3:**
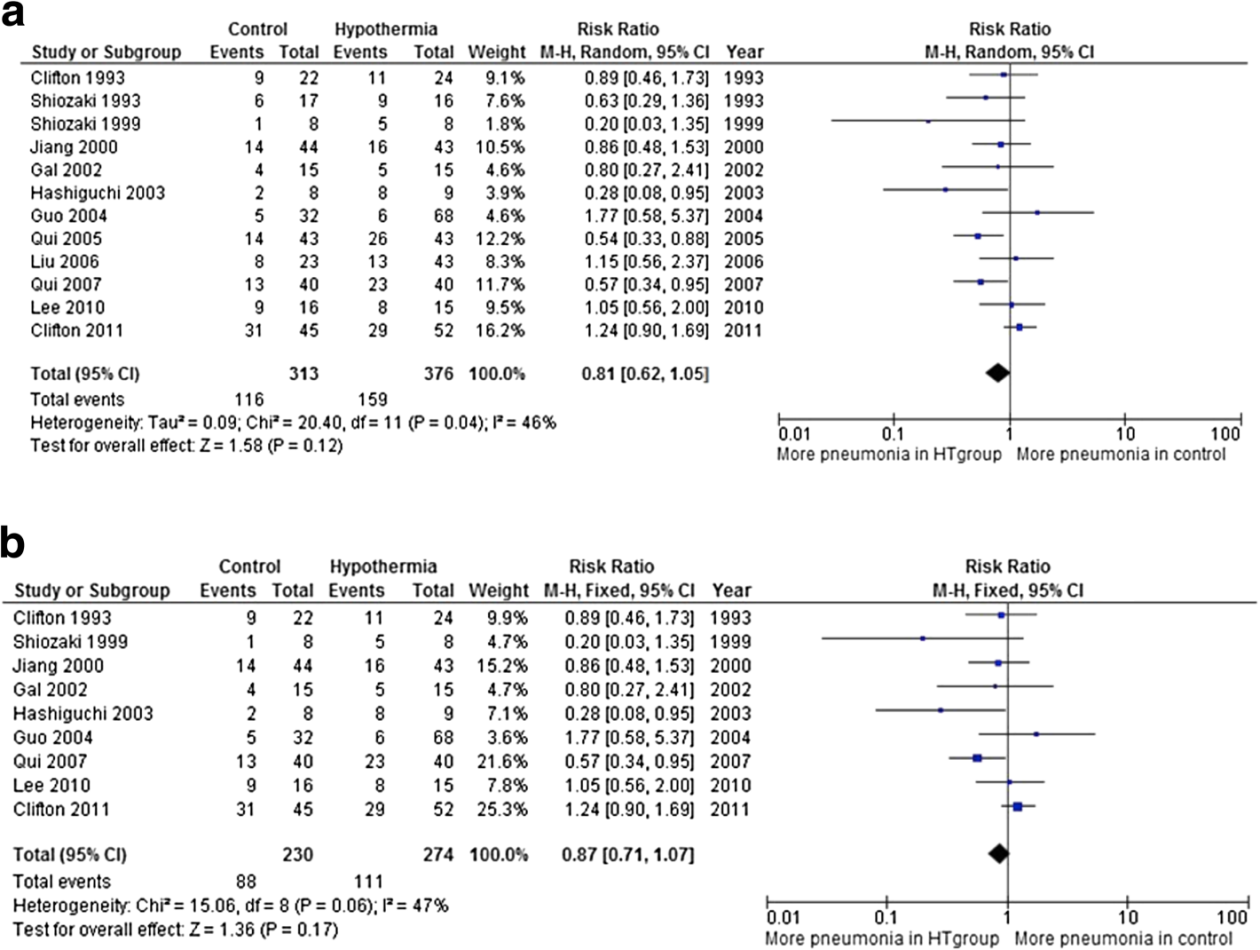
**Incidence of pneumonia during the course of treatment. (a)** In total 12 trials involving 689 patients reported pneumonia in patients during the course of treatment. When the data from 12 randomised controlled trials (RCTs) were aggregated, therapeutic hypothermia had no effect on increasing onset of new pneumonia (relative risk (RR) = 0.81, 95% CI = 0.62, 1.05, *P* = 0.12). **(b)** Trials assessed as lower risk of bias: 9 trials, involving 504 patients, assessed as having lower risk of bias (domain-based assessment) were included in this analysis. When the data from the 9 RCTs that reported pneumonia were aggregated, therapeutic hypothermia had no effect on increasing onset of new pneumonia (RR = 0.87, 95% CI = 0.71, 1.07, *P* = 0.17).

In analysis of trials that reported the incidence of pneumonia during the course of treatment: 12 trials, involving 689 patients, reported pneumonia in patients during the course of treatment. When the data from 12 RCTs were aggregated, therapeutic hypothermia had no effect on increasing onset of new pneumonia. (RR = 0.81, 95% CI = 0.62, 1.05, *P* = 0.12). As the Q test demonstrated that there was evidence of statistical heterogeneity between the trials (*P* = 0.04), and the heterogeneity index suggested a moderate degree of between-study heterogeneity (*I*^2^ = 46%) a random effects model was used (Figure 3).

**Figure 4:**
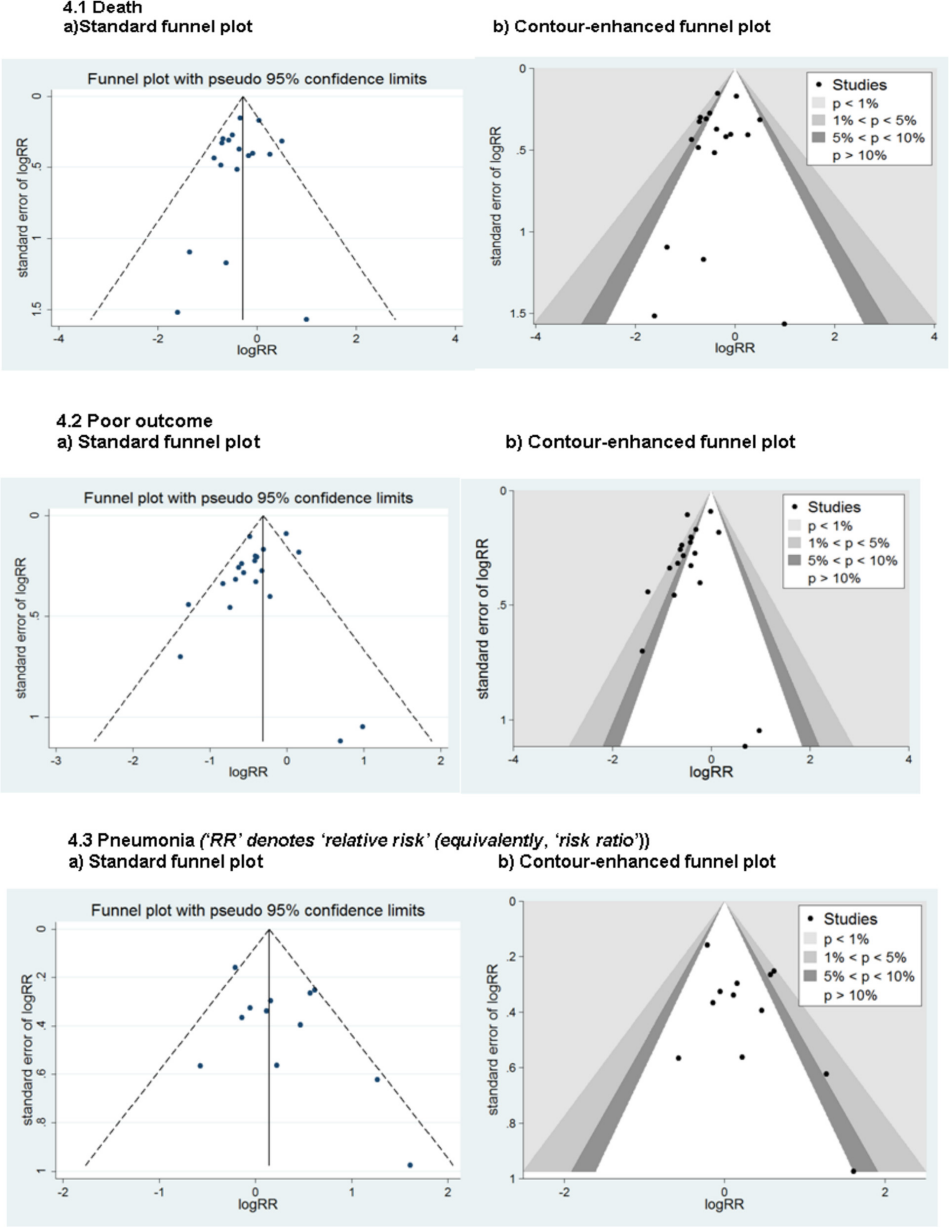
**Funnel plots to investigate evidence of bias.** (**4.1**) Death. **(a)** Standard funnel plot. **(b)** Contour-enhanced funnel plot. (**4.2**) Poor outcome. **(a)** Standard funnel plot. **(b)** Contour-enhanced funnel plot. (**4.3**) Pneumonia; RR, relative risk (equivalently, risk ratio). **(a)** Standard funnel plot. **(b)** Contour-enhanced funnel plot.

Incidence of pneumonia during the course of treatment; analysis of trials assessed as lower risk of bias. Nine trials, involving 504 patients, assessed as lower risk of bias (domain-based assessment) were included in this analysis. When the data from the nine RCTs that reported pneumonia were aggregated, therapeutic hypothermia had no effect on increasing onset of new pneumonia (RR = 0.87, 95% CI = 0.71, 1.07, *P* = 0.17). Although, the *I*^2^ value of 47% was suggestive of a moderate degree of between-study heterogeneity, the *Q*-test revealed a lack of statistical evidence for this type of heterogeneity (χ^2^ = 15.06, *P* = 0.06, df = 8), and a fixed effects model was selected. A random effects model was generated and the conclusions were unaffected (Figure 3b). Standard and contour-enhanced funnel plots for the three outcome categories death, poor outcome and pneumonia are included in Figure 4.

On applying the Begg test (B) and the Egger test (E) there was a lack of evidence for a small-study effect for any one of the principal treatment outcomes, death (B: *P* = 0.940, E: *P* = 0 · 353, n = 18), poor outcome (B: *P* = 0.496 E: *P* = 0.083, n = 20), and pneumonia (B: *P* = 0 · 837, E: *P* = 0.152, n = 12).

### Meta-regression

Using β and the Knapp-Hartung-adjusted regression coefficient (adjR^2^) to denote the regression coefficient and the adjR^2^, respectively, the results of the meta-regression analysis with study risk of bias may be summarized as follows for each of the principal treatment outcomes: for death, β = -0.612, adjR^2^ = 0.612, *P* = 0.065, n = 18; for poor outcome, β = -0.470, adjR^2^ = 0.422, *P* = 0.036, n = 20; and for pneumonia: β = -1.50, adjR^2^ = 1.00, *P* = 0.018, n = 12.

## Discussion

### Summary of results

This systematic review shows there is evidence that therapeutic hypothermia may be beneficial in the treatment of TBI. In the 20 trials included in this systematic review, treatment with therapeutic hypothermia resulted in significantly reduced rates of death, vegetative state, and long-term disability. On removal of the trials with greatest risk of bias, there was an increase in the RR for death (1.31 versus 1.62) and an increase in RR for poor outcome (1.41 to 1.67). Removal of the trials assessed at greatest risk of bias increased the summary estimate of effect. However, caution is recommended as the majority of trials found were of low quality, with unclear allocation concealment. The results indicate that there is a lack of statistical evidence to indicate that treatment with hypothermia has an increased risk of pneumonia.

### Quality of evidence

Therapeutic hypothermia for TBI has been subject to a number of trials of varying size and quality, however, some more recent trials, such as Marion [25] and Clifton [29], have been of better methodological quality. The outcome of a domain-based assessment demonstrated that there are a number of low-quality trials. The impact of these trial data were assessed in a sensitivity analysis, by the exclusion of the trials at highest risk of bias from additional statistical analysis. Although statistical power was reduced, the relative risk remained significant suggesting consistency in outcomes. All selected trials were randomised control trials, however, even in those that met the inclusion criteria the majority (see Additional file 5) had poor explanation of their allocation concealment and/or randomisation methods were vague.

### Limitations and potential bias in the review process

#### Deviation from protocol

There were insufficient data to fully analyse the secondary outcomes; duration of cooling, re-warming rate, and cooling target temperature. All trials universally stated that they cooled patients to 35°C or below and all induced cooling within 6 hours of injury.

#### Limitations of the review process

Due to variations in trial protocol there was variation in the length of time to long-term follow-up, usually between 3 and 6 months. Due to the limited number of trials and trial participants the authors were unable to control for this as a confounding factor.

This systematic review addresses a clear set of primary outcomes using pre-determined inclusion and exclusion criteria, and selection methodology as set out in a publicly available protocol. The pre-published protocol combined with thorough searching and data analysis has reduced the selection risk of bias.

The standard funnel plots (Figure 4) for each of the three main outcomes are designed to display increasing levels of accuracy in estimation of effect size in moving up the y-axis. One can see therefore that for all three outcomes, the majority of trials lend themselves to more accurate estimates, although this is true to a lesser extent in the case of pneumonia. Typically, publication bias would be reflected in a relatively high proportion of trials in the bottom right corner of the plot, due to smaller trials being withheld where the effect was not in the desired direction. However, in these cases, the overall lack of trials with low accuracy is unsupportive of this source of bias or indeed, the more plausible idea that smaller trials tended to produce inflated effect size estimates in the desired direction. The lack of statistical significance for all three principal treatment outcomes following application of the Begg and the Egger tests was supportive of these findings. Nevertheless, it is important to bear in mind that in the presence of a modest number of trials, as in the current review, these tests have limited statistical power in detecting bias. Similar conclusions apply in the case of pneumonia, although here, the loss of trials due to non-events on both arms of a trial would have further reduced the statistical power of the corresponding tests of bias.

The contour-enhanced funnel plot serves as a useful tool in assessing evidence for reported trials being selected according to level of statistical significance, whether through publication bias or through bias arising during the conduct of this study. Only in the case of poor outcome does there appear to be a clustering of this particular sort in the data (Figure 4 4.2b) Although this clustering is around favourable outcomes and in proximity to the region of statistical significance, the absence of this phenomenon for death alone is noteworthy (compare to Figure 4 4.1b). In particular, it is possible that the merging of multiple outcomes in defining poor outcome and the corresponding increase in statistical power afford legitimate reasons for the particular clustering effect for poor outcome. To assess the accuracy of this hypothesis, however, larger RCTs are necessary to support consideration of relative risks for more specific levels of severity of outcome.

The exploratory meta-regression analyses reveal a negative relationship between effect size estimate and study risk of bias. This is true for each of the principal outcomes death, poor outcome, and pneumonia. These results also reveal that this relationship was statistically significant in the case of poor outcome and pneumonia and fairly close to achieving statistical significance in the case of death. Thus, there is good reason to regard the possible beneficial effects of therapeutic hypothermia in preventing death or poor outcome more tentatively. Such findings highlight the need for better-quality trials to provide more reliable evidence in identifying the potential benefits and harms arising from therapeutic hypothermia in adults following TBI.

The above findings from the assessment of study bias can inform this process. Precisely, if, as would seem to be the case for the studies considered here, sample size does not have a dominant role to play in explaining possible relationships between effect size estimate and study quality, and other components of the domain-based assessment also need to be taken carefully into consideration during the trial design process.

### Similarities and differences to other reviews

This systematic review differs from previous reviews as analysis of the 20 studies included in the final selection indicates a statistically significant benefit in the use of therapeutic hypothermia for TBI. The strength of our meta-analysis is based on the fact that we have used several methods to reduce bias (comprehensive literature search, duplicate data abstraction, specific criteria for searching and analysis, risk of bias assessment), have focused on clinically important primary outcomes, and crucially, have excluded trials that include pediatric patients. The previous Cochrane reviews included trials with paediatric patients, however as noted by Adelson *et al*. [12] TBI in the paediatric population involves ‘unique mechanisms, pathophysiologic sequalae, and resultant poor outcomesʼ, potentially causing an altered response to the use of therapeutic hypothermia. Additionally, outcome measures in paediatric patients require careful consideration and are different from those used in adult assessment. Previous reviews have included trials where the functional recovery was assessed by another scale or system than GOS. This review shows results of trials with specific and standardised neurological outcome assessment.

All international trials were included in this review providing they met the selection criteria, whereas other reviews have chosen not to include these trials. Where there was no justifiable reason to remove trials from regions that have been criticized for equivocal methodology and methodology reporting (for example, China), the trials were included. Neither the meta-regression nor the funnel plots showed that this resulted in trial selection bias. The authors recognise that a thorough assessment of trial quality (particularly, risk of bias) as an important factor and therefore utilised a Cochrane domain-based evaluation tool, that was expanded to include other sources of bias that are relevant only in certain circumstances. In particular hypothermia trials can be subject to bias in the way the control group is managed (see Additional files 5 and 6). A table detailing the differences between this systematic review and the previous Cochrane review [9,15] can be found in Additional file 8.

### Ongoing trials of therapeutic hypothermia for traumatic brain injury

There are three ongoing or not yet reported trials. This review identified 20 trials of predetermined quality that recruited only adult patients. All 20 trials studied early hypothermia (within 6 hours of injury) to determine if there was evidence for prophylactic therapeutic hypothermia providing neuroprotection after TBI. There is one ongoing trial, the Prophylactic Hypothermia Trial to Lessen Traumatic Brain Injury (POLAR)-RCT [30] (interim analysis in 2013 and due to report in 2014) and one trial that has not yet been reported, Therapeutic Hypothermia for Severe Traumatic Brain Injury in Japan (BIHYPO) [31] (closed due to futility of recruitment), that both further evaluate prophylactic therapeutic hypothermia after TBI. Data from BIHYPO were kindly provided by the investigator, Professor Maekawa in abstract form only and included in this review.

Commonly, therapeutic hypothermia after TBI is used to reduce raised intracranial pressure (ICP). As yet, no study has investigated if titrated therapeutic hypothermia for raised ICP after TBI reduces death and disability. The Eurotherm3235Trial [32] (due to report in 2017) is a pragmatic trial that is funded by The National Institute for Health Research Health Technology Assessment (NIHR HTA) Programme and will contribute to addressing this important hypothesis.

## Conclusions

This systematic review shows that there may be reduced rates of death and long-term disability among adult patients who receive therapeutic hypothermia treatment following TBI. The results indicate that there is a lack of evidence to suggest that patients treated with therapeutic hypothermia have an increased risk of pneumonia. The authors recognise that therapeutic hypothermia trials pose challenges in recruitment. However there is a need for more high-quality, multicentre, RCTs. Additional work is needed to provide more robust evidence for the benefits and harms before therapeutic hypothermia becomes a widely adopted treatment strategy following TBI. However, the authors believe that therapeutic hypothermia must not be disregarded in the treatment of TBI in adults.

## Key messages

•This systematic review shows there is evidence that therapeutic hypothermia may be beneficial in the treatment of TBI.

•The results indicate that there is a lack of statistical evidence to indicate that treatment with hypothermia has an increased risk of pneumonia when used after TBI.

•The majority of trials were of low quality, with unclear allocation concealment.

•The exploratory meta-regression analyses reveal a negative relationship between effect size estimate and study risk of bias for each of the principal outcomes death, poor outcome, and pneumonia.

•There remains a need for more, high quality, RCTs of therapeutic hypothermia after TBI.

## Abbreviations

adjR2: Knapp-Hartung-adjusted regression coefficient; B: Begg test; BIHYPO: Therapeutic Hypothermia for Severe Traumatic Brain Injury in Japan; CENTRAL: Cochrane Central Register of Controlled Trials; CPCI-S: Conference Proceedings Citation Index-Science; df: degrees of freedom; E: Egger test; Eurotherm3235Trial: European study of therapeutic hypothermia (32 to 35°C) for ICP reduction after traumatic brain injury; GOS: Glasgow outcome scale; I2 index: statistical test assessing heterogeneity in meta-analysis; ICP: intracranial pressure; NICE: National Institute for Health and Clinical Excellence; NIHR HTA: The National Institute for Health Research Health Technology Assessment; PO: poor outcome assessed by the Glasgow outcome scale or similar; POLAR-RCT: The Prophylactic Hypothermia Trial to Lessen Traumatic Brain Injury; Q-test: statistical test to identify statistical outliers in data; RCT: randomised controlled trial; RevMan: Review Manager is the software used for preparing and maintaining Cochrane Reviews; RR: relative risk; RRoverall: overall relative risk; SCI-EXPANDED: Science Citation Index Expanded; Stata: data analysis and statistical software; TBI: traumatic brain injury; TH: therapeutic hypothermia; ZETOC: monitoring and search service for global research publications; β: regression coefficient; χ2: Chi square test.

## Competing interests

Samantha Crossley, Jenny Reid, Rachel McLatchie, Judith Hayton, Clair Clark, Margaret MacDougall declare no competing interests. Peter Andrews: Chief Investigator of the Eurotherm3235 trial, in receipt of funding from National Institute of Health Research, Health Technology Assessment program.

## Authors’ contributions

All authors (SC, JR, PJDA, MM, CC, JH, RM) made substantial contributions to the conception of the work, the acquisition, analysis, and interpretation of data for the work; drafting the work and revising it critically for important intellectual content and final approval of the version to be published.

## Supplementary Material

Additional file 1 Search protocols for the systematic review. Click here for file

Additional file 2 References excluded at the final sift. Click here for file

Additional file 3 PRISMA flow diagram showing the systematic review process. Click here for file

Additional file 4 Domain-based assessment of risk of bias. Click here for file

Additional file 5 Domain-based assessment of risk of bias. Click here for file

Additional file 6 Characteristics of all selected trials. Click here for file

Additional file 7 Prospero protocol for this systematic review. Click here for file

Additional file 8 Differences between this systematic review and the most recent Cochrane review. Click here for file
