# Supplementary material for: A systematic review of therapeutic hypothermia for adult patients following traumatic brain injury
Source: Crit Care. 2014 Apr 17;18(2):R75. doi: 10.1186/cc13835 (PMC4056614; doi:10.1186/cc13835)
Supplement: Additional file 1 — Search protocols for the systematic review. [file cc13835-S1.pdf]

1. Search protocols

Databases should include:

- CENTRAL (*The Cochrane Library*);
- MEDLINE
- PubMed
- EMBASE
- ISI Web of Science: Science Citation Index Expanded (SCI-EXPANDED) and Conference Proceedings Citation Index-Science (CPCI-S);
- Zetoc

Searches Performed 5th Jan 2012

Cochrane Central Register of Controlled Trials (Central)

#1 MeSH descriptor **Craniocerebral Trauma** explode all trees

#2 MeSH descriptor **Brain Edema** explode all trees

#3 MeSH descriptor **Glasgow Coma Scale** explode all trees

#4 MeSH descriptor **Unconsciousness** explode all trees

#5 MeSH descriptor **Glasgow Outcome Scale** explode all trees

#6 MeSH descriptor **Cerebrovascular Trauma** explode all trees

#7 MeSH descriptor **Intracranial Hypertension** explode all trees

#8 (head or crani* or cerebr* or capitis or brain* or forebrain* or skull* or hemispher* or intra-cran* or inter-cran*) near3 (injur* or trauma* or damag* or wound* or fracture* or contusion* or concuss* or pressure*) in Clinical Trials

#9 (head or crani* or cerebr* or capitis or brain* or forebrain* or skull* or hemispher* or intra-cran* or inter-cran* or tentori*) near3 (haematoma* or hematoma* or haemorrhag* or hemorrhag* or bleed* or hernia* or oedema* or edema* or swell*) in Clinical Trials

#10 (Glasgow near3 (coma or outcome) near3 (scale or score)) in Clinical Trials

#11 "Rancho Los Amigos Scale" in Clinical Trials

#12 (diffuse near3 axonal near3 injur*) in Clinical Trials

#13 (unconscious* or coma* or concuss* or 'persistent vegetative state') near3 (injur* or trauma* or damag* or wound* or fracture*) in Clinical Trials

#14 (#1 OR #2 OR #3 OR #4 OR #5 OR #6 OR #7 OR #8 OR #9 OR #10 OR #11 OR #12 OR #13)

#15 MeSH descriptor **Hypothermia, Induced** explode all trees

#16 MeSH descriptor **Cryotherapy** explode all trees

#17 MeSH descriptor **Hypothermia** explode all trees

#18 (hypotherm* or normotherm* or cool* or cold* or temperature* or cryother* or cryogen* or cryotreat*) in Clinical Trials

#19 (refrigeration* or cryo*) near3 anaesthes* in Clinical Trials

#20 (cool* or cold*) near3 (therap* or device* or equipment*) in Clinical Trials

#21 temperature near3 (reduc* or low*) in Clinical Trials

#22 intravenous near3 (cold* or cool*) near3 (fluid* or catheter*) in Clinical Trials

#23 (cool* or cold*) near3 (blanket* or neck collar* or helmet* or hood*) in Clinical Trials

#24 (#15 OR #16 OR #17 OR #18 OR #19 OR #20 OR #21 OR #22 OR #23)

#25 (#14 AND #24)

#26 (#25)

Search protocol produced 432 results

MEDLINE

1. exp Craniocerebral Trauma/
2. exp Brain Edema/
3. exp Glasgow Coma Scale/
4. exp Glasgow Outcome Scale/
5. exp Unconsciousness/
6. exp Cerebrovascular Trauma/
7. exp Intracranial Hypertension/
8. ((head or crani$ or cerebr$ or capitis or brain$ or forebrain$ or skull$ or hemispher$ or intra-cran$ or inter-cran$) adj3 (injur$ or trauma$ or damag$ or wound$ or fracture$ or contusion$ or concuss$ or pressure$)).ab,ti.
9. ((head or crani$ or cerebr$ or capitis or tentori$ or brain$ or forebrain$ or skull$ or hemispher$ or intra-cran$ or inter-cran$) adj3 (haematoma$ or hematoma$ or haemorrhag$ or hemorrhag$ or bleed$ or hernia$ or oedema$ or edema$ or swell$)).ab,ti.
10. (Glasgow adj3 (coma or outcome) adj3 (scale$ or score$)).ab,ti.
11. Rancho Los Amigos Scale.mp.
12. "diffuse axonal injur$".ab,ti.
13. ((unconscious$ or coma$ or concuss$ or 'persistent vegetative state') adj3 (injur$ or trauma$ or damag$ or wound$ or fracture$)).ab,ti.
14. or/1-13
15. exp Hypothermia, Induced/
16. exp Cryotherapy/
17. exp Hypothermia/
18. (hypotherm$ or normotherm$ or cool$ or cold$ or temperature$ or cryother$ or cryogen$ or cryotreat$).ab,ti.
19. ((refrigeration$ or cryo$) adj3 anaesthes$).ab,ti.
20. ((cool$ or cold$) adj3 (therap$ or device$ or equipment$)).ab,ti.
21. (temperature adj3 (reduc$ or low$)).ab,ti.
22. (intravenous adj3 (cold$ or cool$) adj3 (fluid$ or catheter$)).ab,ti.
23. ((cool$ or cold$) adj3 (blanket$ or neck collar$ or helmet$ or hood$)).ab,ti.
24. or/15-23
25. (randomised or randomized or randomly or random order or random sequence or random allocation or randomly allocated or at random or controlled clinical trial$).tw,hw.
26. clinical trial.pt.
27. randomized controlled trial.pt.
28. or/25-27
29. exp models, animal/
30. exp Animals/
31. exp Animal Experimentation/
32. exp Animals, Laboratory/
33. or/29-32
34. Humans/
35. 33 not 34
36. 28 not 35
37. 36 and 14 and 24

Search protocol produced 477 results

PubMed

1. **Craniocerebral Trauma[MeSH Terms] OR Brain Edema[MeSH Terms] OR Glasgow Coma Scale[MeSH Terms] OR Glasgow Outcome Scale[MeSH Terms] OR Unconsciousness[MeSH Terms] OR Cerebrovascular Trauma[MeSH Terms] OR Intracranial hypertension[MeSH Terms]**
2. **(head OR cranial OR cerebral OR capitis OR brain OR forebrain* OR skull* OR hemispher* OR intra-cran* OR inter-cran*) AND (injur* OR trauma OR damag* OR wound* OR fracture* OR contusion* OR concuss* OR pressure*)**
3. **(head OR cranial OR cerebral OR capitis OR brain OR forebrain* OR skull* OR hemispher* OR intra-cran* OR inter-cran* OR tentori*) AND (haematoma* OR hematoma* OR haemorrhag* OR hemorrhag* OR bleed* OR hernia* OR oedema* OR edema* or swell*)**
4. **glasgow AND (scale OR score) AND (outcome OR coma)**
5. **Ranchos Los Amigos**
6. **diffuse axonal injur***
7. **(unconscious OR coma* OR concuss* OR persistent vegetative state) AND (injur* OR trauma OR damag* OR wound* OR fracture*)**
8. **COMBINE**
9. **Hypothermia, Induced[MeSH Terms] OR Cryotherapy[MeSH Terms] OR Hypothermia[MeSH Terms]**
10. **hypotherm* OR normotherm* OR cool* OR cold* OR temperature* OR cryother* OR cryogen* OR cryotreat***
11. **((refrigeration* OR cryo*) AND anaesthes*) OR ((cool* OR cold*) AND (therap* OR device* OR equipment*)) OR ((temperature AND (reduc* OR low*)) OR ((intravenous AND (cold* OR cool*) AND (fluid* OR catheter*)) OR ((cool* OR cold*) AND (blanket* OR neck collar* OR helmet* OR hood*))**
12. **COMBINE**
13. **(randomised OR randomized OR randomly OR random order OR random sequence OR random allocation OR randomly allocated OR at random OR randomized controlled trial [pt] OR controlled clinical trial [pt] OR randomized controlled trials[MeSH Terms]) NOT ((models, animal[MeSH Terms] OR Animals[MeSH Terms] OR Animal Experimentation[MeSH Terms] OR Disease Models, Animal[MeSH Terms] OR Animals, Laboratory[MeSH Terms]) NOT (Humans[MeSH Terms]))**
14. **COMBINE**

(can also be written and input as protocol below)
((Craniocerebral Trauma[MeSH Terms] OR Brain Edema[MeSH Terms] OR Glasgow Coma Scale[MeSH Terms] OR Glasgow Outcome Scale[MeSH Terms] OR Unconsciousness[MeSH Terms] OR Cerebrovascular Trauma[MeSH Terms] OR Intracranial hypertension[MeSH Terms]) OR ((head OR cranial OR cerebral OR capitis OR brain OR forebrain* OR skull* OR hemispher* OR intra-cran* OR inter-cran*) AND (injur* OR trauma OR damag* OR wound* OR fracture* OR contusion* OR concuss* OR pressure*)) OR ((head OR cranial OR cerebral OR capitis OR brain OR forebrain* OR skull* OR hemispher* OR intra-cran* OR inter-cran* OR tentori*) AND (haematoma* OR hematoma* OR haemorrhag* OR hemorrhag* OR bleed* OR hernia* OR oedema* OR edema* OR swell*)) OR (glasgow AND (scale OR score) AND (outcome OR coma)) OR (Ranchos Los Amigos) OR (diffuse axonal injur*) OR ((unconscious OR coma* OR concuss* OR persistent vegetative state) AND (injur* OR trauma OR damag* OR wound* OR fracture*))) AND ((Hypothermia, Induced[MeSH Terms] OR Cryotherapy[MeSH Terms] OR Hypothermia[MeSH Terms]) OR (hypotherm* OR normotherm* OR cool* OR cold* OR temperature* OR cryother* OR cryogen* OR cryotreat*) OR (((refrigeration* OR cryo*) AND anaesthes*) OR ((cool* OR cold*) AND (therap* OR device* OR equipment*)) OR (temperature AND (reduc* OR low*)) OR (intravenous AND (cold* OR cool*) AND (fluid* OR catheter*)) OR ((cool* OR cold*) AND (blanket* OR neck collar* OR helmet* OR hood*)))) AND ((randomised OR randomized OR randomly OR random order OR random sequence OR random allocation OR randomly allocated OR at random OR randomized controlled trial[pt] OR controlled clinical trial[pt] OR randomized controlled trials[MeSH Terms]) NOT ((models, animal[MeSH Terms] OR Animals[MeSH Terms] OR Animal Experimentation[MeSH Terms] OR Disease Models, Animal[MeSH Terms] OR Animals, Laboratory[MeSH Terms]) NOT (Humans[MeSH Terms])))
Search protocol produced 806 results

EMBASE

1. exp Craniocerebral Trauma/
2. exp Brain Edema/
3. exp Glasgow Coma Scale/
4. exp Glasgow Outcome Scale/
5. exp Unconsciousness/
6. exp Cerebrovascular Trauma/
7. exp Intracranial Hypertension/
8. ((head or crani$ or cerebr$ or capitis or brain$ or forebrain$ or skull$ or hemispher$ or intra-cran$ or inter-cran$) adj3 (injur$ or trauma$ or damag$ or wound$ or fracture$ or contusion$ or concuss$ or pressure$)).ab,ti.
9. ((head or crani$ or cerebr$ or capitis or tentori$ or brain$ or forebrain$ or skull$ or hemispher$ or intra-cran$ or inter-cran$) adj3 (haematoma$ or hematoma$ or haemorrhag$ or hemorrhag$ or bleed$ or hernia$ or oedema$ or edema$ or swell$)).ab,ti.
10. (Glasgow adj3 (coma or outcome) adj3 (scale$ or score$)).ab,ti.
11. exp Rancho Los Amigos Scale/
12. "diffuse axonal injur$".ab,ti.
13. ((unconscious$ or coma$ or concuss$ or 'persistent vegetative state') adj3 (injur$ or trauma$ or damag$ or wound$ or fracture$)).ab,ti.
14. or/1-13
15. exp Induced Hypothermia/
16. exp Cryotherapy/
17. exp Hypothermia/
18. exp Profound Induced Hypothermia/
19. (hypotherm$ or normotherm$ or cool$ or cold$ or temperature$ or cryo$).ab,ti.
20. ((refri?geration$ or cryo$) adj3 anaesthes$).ab,ti.
21. ((cool$ or cold$) adj3 (therap$ or device$ or equipment$)).ab,ti.
22. (temperature adj3 (reduc$ or low$)).ab,ti.
23. (intravenous adj3 (cold$ or cool$ or ice$ or refrigerat$) adj3 (fluid$ or catheter$)).ab,ti.
24. ((cool$ or cold$) adj3 (blanket$ or cap$ or pad$ or neck collar$ or helmet$ or hood$)).ab,ti.
25. or/15-24
26. (randomised or randomized or randomly or random order or random sequence or random allocation or randomly allocated or at random or controlled clinical trial$).tw,hw.

27. exp Clinical Trial/
28. randomised control trial.pt.
29. or/26-28
30. exp Animal Models/
31. exp Animals/
32. exp Animal Experimentation/
33. exp Laboratory Animals/
34. or/30-33
35. Humans/
36. 34 not 35
37. 29 not 36
38. 37 and 14 and 25

Search protocol produced 1210 results

ISI Web of Science: Science Citation Index Expanded (SCI-EXPANDED) and Conference Proceedings Citation Index-Science (CPCI-S)

#1 Topic=(head OR crani* OR capitis OR brain* OR forebrain* OR skull* OR hemisphere* OR intracran* OR intercran) AND Topic=(injur* OR trauma* OR lesion* OR damag* OR wound* OR haematoma* OR oedema* OR edema* OR fracture* OR contusion* OR concus* OR commotion* OR pressur*) AND Topic=(hypotherm* OR normotherm* OR cool* OR cold* OR temperature* OR cryother* OR cryogen* or cryotreat)
Databases=SCI-EXPANDED, CPCI-S Timespan=1960-2011
Lemmatization=On

#2 Topic=(randomised OR randomized OR randomly OR random order OR random sequence OR random allocation OR randomly allocated OR at random OR randomized controlled trial OR controlled clinical trial OR randomized controlled trials OR controlled trial OR clinical trial) NOT Topic=(animal model* OR Animals OR Animal Experiment* OR Laboratory animal* OR animal disease model*)
Databases=SCI-EXPANDED, CPCI-S Timespan=1960-2011
Lemmatization=On

#3 #2 AND #1
Databases=SCI-EXPANDED, CPCI-S Timespan=1960-2011
Lemmatization=On

Search protocol produced 955 results

<u>Zetoc</u>
Hypotherm* head injur* trial*
Hypotherm* head injur* random*
Hypotherm* head injur* control*

Hypotherm* brain injur* trial*
Hypotherm* brain injur* random*
Hypotherm* brain injur* control*

Hypotherm* head trauma* trial*
Hypotherm* head trauma* random*
Hypotherm* head trauma* control*

Hypotherm* brain trauma* trial*
Hypotherm* brain trauma* random*
Hypotherm* brain trauma* control*

Search protocol produced 547 results
