# Supplementary material for: A systematic review of therapeutic hypothermia for adult patients following traumatic brain injury
Source: Crit Care. 2014 Apr 17;18(2):R75. doi: 10.1186/cc13835 (PMC4056614; doi:10.1186/cc13835)
Supplement: Additional file 2 — References excluded at the final sift. [file cc13835-S2.docx]

**References for trials excluded at the final sift:**

1. Andrews PJ, Sinclair HL, Battison CG, Polderman KH, Citerio G, Mascia L, et al. European society of intensive care medicine study of therapeutic hypothermia (32-35 degreeC) for intracranial pressure reduction after traumatic brain injury (the Eurotherm3235Trial). Trials. 2011;12:8.
2. Bayir H, Marion DW, Puccio AM, Wisniewski SR, Janesko KL, Clark RSB, et al. Marked gender effect on lipid peroxidation after severe traumatic brain injury in adult patients. Journal of Neurotrauma. 2004 Jan;21(1):1-8.
3. Bernard SA, MacC Jones B, Buist M. Experience with prolonged induced hypothermia in severe head injury. Critical Care. 1999;3(6):167-72.
4. Carhuapoma JR, Gupta K, Coplin WM, Muddassir SM, Meratee MM. Treatment of refractory fever in the neurosciences critical care unit using a novel, water-circulating cooling device. A single-center pilot experience. Journal of Neurosurgical Anesthesiology. 2003 Oct;15(4):313-8.
5. Chen L, Piao Y, Zeng F, Lu M, Kuang Y, Ki X. Moderate hypothermia therapy for patients with severe head injury. Chinese Journal of Traumatology. 2001 Aug;4(3):164-7.
6. Cheng SX, Shen YF, Zhang S. The effect of moderate hypothermia on amyloid-beta in cerebrospinal fluid after severe traumatic brain injury. Journal of Neurotrauma. 2011;28 (5):A86.
7. Chi JH. Craniectomy for traumatic brain injury: Results from the DECRA Trial. Neurosurgery. 2011 June;68(6):N19-N20.
8. Clark RS, Kochanek PM, Obrist WD, Wong HR, Billiar TR, Wisniewski SR, et al. Cerebrospinal fluid and plasma nitrite and nitrate concentrations after head injury in humans. Critical Care Medicine. 1996 Jul;24(7):1243-51.
9. Clifton GL, Allen S, Barrodale P, Plenger P, Berry J, Koch S, Fletcher J, Hayes RL, Choi SC. A phase II study of moderate hypothermia in severe brain injury. Journal of Neurotrauma. 1994; (3): Available from: <http://www.mrw.interscience.wiley.com/cochrane/clcentral/articles/864/CN-00407864/frame.html>.
10. Clifton GL. Hypothermia and hyperbaric oxygen as treatment modalities for severe head injury. New Horizons. 1995 Aug;3(3):474-8.
11. Clifton GL. Systemic hypothermia in treatment of severe brain injury. Journal of Neurosurgical Anesthesiology. 1995 Apr;7(2):152-6.
12. Clifton GL. Hypothermia in neurotrauma. 3rd International Neurotrauma Symposium [serial on the Internet]. 1995: Available from: <http://www.mrw.interscience.wiley.com/cochrane/clcentral/articles/490/CN-00690490/frame.html>.
13. Clifton GL. Development and status of hypothermia for brain injury: National Acute Brain Injury Study: Hypothermia. Hayashi N, editor 2000.
14. Clifton GL, Allen S, Berry J, Koch SM. Systemic hypothermia in treatment of brain injury. Journal of Neurotrauma. 1992 May;9 Suppl 2:S487-95.
15. Clifton GL, Drever P, Valadka A, Zygun D, Okonkwo D. Multicenter trial of early hypothermia in severe brain injury. Journal of Neurotrauma. 2009 Mar;26(3):393-7.
16. Clifton GL, Knudson P, McDonald M. Waiver of consent in studies of acute brain injury. Journal of Neurotrauma. 2002 Oct;19(10):1121-6.
17. Clifton GL, Miller ER, Choi SC, Levin HS. Fluid thresholds and outcome from severe brain injury. Critical Care Medicine. 2002 Apr;30(4):739-45.
18. Clifton GL, Miller ER, Choi SC, Levin HS, McCauley S, Smith Jr KR, et al. Hypothermia on admission in patients with severe brain injury. Journal of Neurotrauma. 2002;19(3):293-301.
19. Diringer MN, Neurocritical Care Fever Reduction Trial G. Treatment of fever in the neurologic intensive care unit with a catheter-based heat exchange system. Critical Care Medicine. 2004 Feb;32(2):559-64.
20. Fernandez A, Schmidt JM, Claassen J, Pavlicova M, Huddleston D, Kreiter KT, et al. Fever after subarachnoid hemorrhage - Risk factors and impact on outcome. Neurology. 2007 Mar;68(13):1013-9.
21. Fischer M, Lackner P, Beer R, Helbok R, Klien S, Ulmer H, et al. Keep the brain cool-endovascular cooling in patients with severe traumatic brain injury: A case series study. Neurosurgery. 2011 April;68(4):867-73.
22. Francony G, Declety P, Bouzat P, Picard J, Payen JF. [Hazards of therapeutic hypothermia]. Annales Francaises d Anesthesie et de Reanimation. 2009 Apr;28(4):371-4.
23. Gal R, Cundrle I, Stibor B. Intracranial and cerebral perfusion pressure changes in deliberate hypothermia in patients with severe brain injury. [Czech]
24. Zmeny intrakranialniho a mozkoveho perfuzniho tlaku v prubehu rizene hypotermie u pacientu s tezkym poranenim mozku. Lekarsky Obzor. 2002;51(6):165-7.
25. Gasser S, Khan N, Yonekawa Y, Imhof HG, Keller E. Long-term hypothermia in patients with severe brain edema after poor-grade subarachnoid hemorrhage: feasibility and intensive care complications. Journal of Neurosurgical Anesthesiology [serial on the Internet]. 2003; (3): Available from: <http://www.mrw.interscience.wiley.com/cochrane/clcentral/articles/885/CN-00558885/frame.html>.
26. Harris OA, Colford JM, Jr., Good MC, Matz PG. The role of hypothermia in the management of severe brain injury: a meta-analysis. Archives of Neurology. 2002 Jul;59(7):1077-83.
27. Harris OA, Muh CR, Surles MC, Pan Y, Rozycki G, Macleod J, et al. Discrete cerebral hypothermia in the management of traumatic brain injury: a randomized controlled trial.[Erratum appears in J Neurosurg. 2009 Jun;110(6):1322]. Journal of Neurosurgery. 2009 Jun;110(6):1256-64.
28. Hartemink KJ, Wisselink W, Rauwerda JA, Girbes AR, Polderman KH. Novel applications of therapeutic hypothermia: report of three cases. Critical Care. 2004;8(5):R343-R6.
29. Hartung J, Marion DW. Head injury and therapeutic hypothermia [4]. Journal of Neurosurgery. 1994;80(4):776.
30. Hayashi S, Takayasu M, Inao S, Yoshida J. Balance of risk of therapeutic hypothermia. Acta Neurochirurgica. 2005;Supplement. 95:269-72.
31. He XM, Mo XM, Gu HT, Chen F, Gu Q, Peng W, et al. Neuroprotective effect of diazoxide on brain injury induced by cerebral ischemia/reperfusion during deep hypothermia. Journal of the Neurological Sciences. 2008 May;268(1-2):18-27.Health Technology A. Systematic review of head cooling in adults after traumatic brain injury and stroke (Project record). Health Technology Assessment [serial on the Internet]. 2010: Available from: <http://www.mrw.interscience.wiley.com/cochrane/clhta/articles/HTA-32010000318/frame.html>.
32. Henderson WR, Dhingra VK, Chittock DR, Fenwick JC, Ronco JJ. Hypothermia in the management of traumatic brain injury. A systematic review and meta-analysis. Intensive Care Medicine. 2003 Oct;29(10):1637-44.
33. Henderson WR, Dhingra VK, Ronco JJ. Lack of effect of hypothermia in the management of traumatic closed head injury. American Journal of Respiratory and Critical Care Medicine [serial on the Internet]. 2002; (Suppl 8): Available from: <http://www.mrw.interscience.wiley.com/cochrane/clcentral/articles/132/CN-00670132/frame.html>.
34. Hernandez AV, Steyerberg EW, Taylor GS, Marmarou A, Habbema JDF, Maas AIR. Subgroup analysis and covariate adjustment in randomized clinical trials of traumatic brain injury: A systematic review. Neurosurgery. 2005 Dec;57(6):1244-53.
35. Himmelseher S. Hypothermia after traumatic brain injury. Report of the Neuroonaesthesia Working Group of the German Society of Anaesthesiology and Intensive Care Medicine. Anasthesiologie & Intensivmedizin. 2004 May;45(5):262-82.
36. Hirayama T, Kano T, Katayama y, Hayashi N, Tsubokawa T. Randomized trial of hypothermia in patients with severe head injury. Journal of Neurotrauma [serial on the Internet]. 1994; (2): Available from: <http://www.mrw.interscience.wiley.com/cochrane/clcentral/articles/665/CN-00369665/frame.html>.
37. Hirayama T, Katayama Y, Maeda T, Kawamata T, Tsubokawa T. Effects Of Moderate Hypothermia On The Evolution Of Cerebral Contusion. 3rd International Neurotrauma Symposium [serial on the Internet]. 1995: Available from: <http://www.mrw.interscience.wiley.com/cochrane/clcentral/articles/667/CN-00369667/frame.html>.
38. Hoedemaekers CW, Ezzahti M, Gerritsen A, van der Hoeven JG. Comparison of cooling methods to induce and maintain normo- and hypothermia in intensive care unit patients: a prospective intervention study. Critical Care. 2007;11(4):R91.
39. Honeybul S, Ho K, Lind C, Gillett G. Hypothermia in patients with brain injury: the way forward? Lancet Neurology. 2011 May;10(5):405-6; author reply 6-7.
40. Hulme J. Resuscitation of patients after traumatic brain injury. Trauma. 2008 Jan;10(1):55-63.
41. Inamasu J, Saito R, Nakamura Y, Horiguchi T, Kuroshima Y, Ichikizaki K. Therapeutic hypothermia for severely head-injured patients with acute subdural haematoma. Journal of Clinical Neuroscience. 2006 Aug;13(7):733-7.
42. Ishikura H, Yamagami K, Akahori M, Shoji Y, Fukui H, Tanaka T. Changes in blood platelet count and serum thrombpoetin (TPO) level under moderate hypothermic therapy in traumatic severe closed head injury. Critical Care Medicine [serial on the Internet]. 1998; (1): Available from: <http://www.mrw.interscience.wiley.com/cochrane/clcentral/articles/689/CN-00369689/frame.html>.
43. Jiang JY. Clinical Study of Mild Hypothermia Treatment for Severe Traumatic Brain Injury. Journal of Neurotrauma. 2009 Mar;26(3):399-406.
44. Jiang JY, Xu W, Li WP, Gao GY, Bao YH, Liang YM, et al. Effect of long-term mild hypothermia or short-term mild hypothermia on outcome of patients with severe traumatic brain injury. Journal of cerebral blood flow and metabolism : official journal of the International Society of Cerebral Blood Flow and Metabolism [serial on the Internet]. 2006; (6): Available from: <http://www.mrw.interscience.wiley.com/cochrane/clcentral/articles/563/CN-00561563/frame.html>.
45. Jiang JY, Yang XF. Current status of cerebral protection with mild-to-moderate hypothermia after traumatic brain injury. Current Opinion in Critical Care. 2007 Apr;13(2):153-5.
46. Johnston NJ, King AT, Protheroe R, Childs C. Body temperature management after severe traumatic brain injury: Methods and protocols used in the United Kingdom and Ireland. Resuscitation. 2006 Aug;70(2):254-62.
47. Kamps M, Bisschops LA, van der Hoeven JG, Hoedemaekers CWE. Hypothermia does not increase the risk of infection: a case control study. Critical Care. 2011;15(1).
48. Keel M, Labler L, Trentz O. "Damage control" in severely injured patients: Why, when, and how? European Journal of Trauma. 2005 Jun;31(3):212-21.
49. Kimberger O, Kurz A. Thermoregulatory management for mild therapeutic hypothermia. Best Practice and Research: Clinical Anaesthesiology. 2008 December;22(4):729-44.
50. Kitsuta Y, Suzuki N, Chang C, Yoshida T, Shuto T, Noguchi T, et al., editors. Hypothermia therapy for severe brain injury. Recent advances in neurotraumatology; 1999 Nov; Taipei: Monduzzi Editore.
51. Koba Y, Miyake Y, Aruga T, Kusaka K, Kato G, editors. Re-examination of hypothermia in the acute phase of a head injury model. Recent advances in neurotraumatology; 1999 Nov; Taipei: Monduzzi Editore.
52. Kochanek PM, Safar PJ. Therapeutic Hypothermia for Severe Traumatic Brain Injury. Journal of the American Medical Association. 2003 11 Jun;289(22):3007-9.
53. Liu W, An YH, Liu EZ, Yu CJ. Effect of mild hypothermia combined with hibernation on the homeostasis of patients with severe head injury. Chinese Journal of Clinical Rehabilitation. 2005 07 Sep;9(33):175-7.
54. Marion Dw CP. Moderate Therapeutic Hypothermia Improves Outcome Following Severe Traumatic Brain Injury. 3rd International Neurotruama Symposium [serial on the Internet]. 1995: Available from: <http://www.mrw.interscience.wiley.com/cochrane/clcentral/articles/819/CN-00690819/frame.html>.
55. Mayer S, Commichau C, Scarmeas N, Presciutti M, Bates J, Copeland D. Clinical trial of an air-circulating cooling blanket for fever control in critically ill neurologic patients. Neurology. 2001 Feb 13;56(3):292-8.
56. McCauley SR, Levin HS, Vanier M, Mazaux JM, Boake C, Goldfader PR, et al. The neurobehavioural rating scale-revised: sensitivity and validity in closed head injury assessment. Journal of Neurology, Neurosurgery & Psychiatry. 2001 Nov;71(5):643-51.
57. Nordby HK, Nesbakken R. The effect of high dose barbiturate decompression after severe head injury. A controlled clinical trial. Acta Neurochirurgica. 1984;72(3-4):157-66.
58. Piepgras A, Roth H, Schurer L, Tillmans R, Quintel M, Herrmann P, et al. Rapid active internal core cooling for induction of moderate hypothermia in head injury by use of an extracorporeal heat exchanger. Neurosurgery. 1998 Feb;42(2):311-7; discussion 7-8.
59. Pierson R, Torlinski T. Early hypothermia induction in patients with severe brain injury. Journal of the Intensive Care Society. 2011 October;12(4):335-7.
60. Pittl U, Schratter A, Klemm T, Lehmann D, Demmin K, Schuler G, et al. Randomized comparison of surface- and invasive cooling after sudden cardiac arrest. European Heart Journal. 2010;31:900.
61. Polderman KH, Joe RTT, Peerdeman SM, Vandertop WP, Girbes ARJ. Effects of therapeutic hypothermia on intracranial pressure and outcome in patients with severe head injury. Intensive Care Medicine. 2002 Nov;28(11):1563-73.
62. Popugaev KA, Savin IA, Goriachev AS, Oshorov AV, Kurdiumova NV, Sychev AA, et al. [First experience of using CoolGard system in intensive care patients after neurosurgical interventions: series of 10 observations]. Anesteziologiia i Reanimatologiia. 2011 Mar-Apr(2):42-9.
63. Qiu WS, Shen H, Zhang Y, Wang WM, Liu WG, Jiang QZ, et al. Noninvasive selective brain cooling by head and neck cooling is protective in severe traumatic brain injury. Journal of Clinical Neuroscience. 2006 Dec;13(10):995-1000.
64. Qiu W-s, Wang W-m, Du H-y, Liu W-g, Shen H, Shen L-f, et al. Thrombocytopenia after therapeutic hypothermia in severe traumatic brain injury. Chinese Journal of Traumatology. 2006 Aug;9(4):238-41.
65. Sahuquillo J, Perez-Barcena J, Biestro A, Zavala E, Merino MA, Vilalta A, et al. Intravascular cooling for rapid induction of moderate hypothermia in severely head-injured patients: results of a multicenter study (IntraCool). Intensive Care Medicine. 2009 May;35(5):890-8.
66. Sai Z, Tu Y, Fan GM. Dynamic changes of serum anti-brain antibody and C-reactive protein in patients with severe traumatic brain injury after mild hypothermia treatment. Journal of Neurotrauma. 2011;28 (5):A76.
67. Sai Z, Tu Y, Sun WC. Decreased soluble Fas levels in cerebrospinal fluid of patients with severe traumatic brain injury after mild hypothermia treatmen. Journal of Neurotrauma. 2011;28 (5):A75.
68. Saur J, Leweling H, Trinkmann F, Weissmann J, Borggrefe M, Kaden JJ. Modification of the Harris-Benedict equation to predict the energy requirements of critically ill patients during mild therapeutic hypothermia. In Vivo. 2008 Jan;22(1):143-6.
69. Scheibel RS, Levin HS, Clifton GL. Completion rates and feasibility of outcome measures: experience in a multicenter clinical trial of systemic hypothermia for severe head injury. Journal of Neurotrauma. 1998 Sep;15(9):685-92.
70. Shafi S, Elliott AC, Gentilello L. Is hypothermia simply a marker of shock and injury severity or an independent risk factor for mortality in trauma patients? Analysis of a large national trauma registry. Journal of Trauma-Injury Infection and Critical Care. 2005 Nov;59(5):1081-5.
71. Shiozaki T, Akai H, Taneda M, Hayakata T, Aoki M, Oda J, et al. Delayed hemispheric neuronal loss in severely head-injured patients. Journal of Neurotrauma. 2001;18(7):665-74.
72. Shiozaki T, Hayakata T, Taneda M, Nakajima Y, Hashiguchi N, Fujimi S, et al. A multicenter prospective randomized controlled trial of the efficacy of mild hypothermia for severely head injured patients with low intracranial pressure. Mild Hypothermia Study Group in Japan. Journal of Neurosurgery. 2001 Jan;94(1):50-4.
73. Shiozaki T, Nakajima Y, Taneda M, Tasaki O, Inoue Y, Ikegawa H, et al. Efficacy of moderate hypothermia in patients with severe head injury and intracranial hypertension refractory to mild hypothermia. Journal of Neurosurgery. 2003 Jul;99(1):47-51.
74. Shiozaki T, Sugimoto H, Taneda M, Oda J, Tanaka H, Hiraide A, et al. Selection of severely head injured patients for mild hypothermia therapy. Journal of Neurosurgery. 1998 Aug;89(2):206-11.
75. Sinz EH, Kochanek PM, Heyes MP, Wisniewski SR, Bell MJ, Clark RS, et al. Quinolinic acid is increased in CSF and associated with mortality after traumatic brain injury in humans. Journal of Cerebral Blood Flow & Metabolism. 1998 Jun;18(6):610-5.
76. Slade J, Kerr ME, Marion D. Effect of therapeutic hypothermia on the incidence and treatment of intracranial hypertension. Journal of Neuroscience Nursing. 1999 Oct;31(5):264-9.
77. Smrcka M, Vidlak M, Maca K, Smrcka V, Gal R. The use of mild hypothermia in the prevention of secondary brain injury. Hypothermia for Acute Brain Injury: Pathomechanism and Practical Aspects. Hayashi N, Bullock R, Dietrich DW, Maekawa T, Tamura A, editors 2004: 251-54.
78. Tateishi A, Soejima Y, Taira Y, Nakashima K, Fujisawa H, Tsuchida E, et al. Feasibility of the Titration Method of Mild Hypothermia in Severely Head-injured Patients with Intracranial Hypertension. Neurosurgery. 1998;42(5):1065-70.
79. Tianan Z, Yinyong Z, Bai H. Clinical analysis of mild hypothermia combined with hyperbaric oxygen for traumatic cerebral infarction. Journal of Neurotrauma. 2011;28 (5):A38.
80. Utagawa A, Sakurai A, Kinoshita K, Moriya T, Hayashi N. Immune-enhancing effect of arginine on patients with severe traumatic brain injuries who have undergone therapeutic brain hypothermia. Hayashi N, Bullock R, Dietrich DW, Maekawa T, Tamura A, editors2004.
81. Utagawa A, Sakurai A, Kinoshita K, Moriya T, Okuno K, Tanjoh K. Organ dysfunction assessment score for severe head injury patients during brain hypothermia. Acta Neurochirurgica - Supplement. 2006;96:33-6.
82. Wagner AK, Bayir H, Ren D, Puccio A, Zafonte RD, Kochanek PM. Relationships between cerebrospinal fluid markers of excitotoxicity, ischemia, and oxidative damage after severe TBI: the impact of gender, age, and hypothermia. Journal of Neurotrauma. 2004 Feb;21(2):125-36.
83. Wagner AK, Fabio A, Puccio AM, Hirschberg R, Li W, Zafonte RD, et al. Gender associations with cerebrospinal fluid glutamate and lactate/pyruvate levels after severe traumatic brain injury. Critical Care Medicine. 2005 Feb;33(2):407-13.
84. Wang Q, Li AL, Zhi DS, Huang HL. Effect of mild hypothermia on glucose metabolism and glycerol of brain tissue in patients with severe traumatic brain injury. Chinese Journal of Traumatology - English Edition. 2007 01 Aug;10(4):246-9.
85. Wang WP, Ren HJ, Chi JY, Xu FL, Quan Y. Effects of mild hypothermia on patients with lower intracranial pressure following severe brain injury. Chinese journal of traumatology/ Chinese Medical Association [serial on the Internet]. 2005; (1): Available from: http://www.mrw.interscience.wiley.com/cochrane/clcentral/articles/855/CN-00513855/frame.html.
86. Xia YQ, Yan LL, Xu RX, Wang QH. Evaluation of improvement of subhypothermia in cerebral vasopasm after severe craniocerebral injury. Chinese Journal of Clinical Rehabilitation. 2005 07 Nov;9(41):138-41.
87. Yan Y, Tang W. Changes of evoked potentials and evaluation of mild hypothermia for treatment of severe brain injury. Chinese Journal of Traumatology. 2001 Feb;4(1):8-13.
88. Yan Y, Tang W, Deng Z, Zhong D, Yang G. Cerebral oxygen metabolism and neuroelectrophysiology in a clinical study of severe brain injury and mild hypothermia. Journal of Clinical Neuroscience. 2010 February;17(2):196-200.
89. Yan Y, Tang WY, He JG, Gao JJ, Dan W, Zhong D, et al. [Clinical research about brain oxygen metabolism and neuroelectrophysiology during mild hypothermia in patients with severe head injury]. Zhonghua wai ke za zhi [Chinese journal of surgery] [serial on the Internet]. 2007; (2): Available from: <http://www.mrw.interscience.wiley.com/cochrane/clcentral/articles/766/CN-00636766/frame.html>.
90. Zhi DS, Zhang S, Chen HH, Zhou LG, editors. Continuous monitoring of brain tissue oxygen pressure in patients with severe head injury during moderate hypothermia. Recent advances in neurotraumatology; 1999 Nov; Taipei: Monduzzi Editore, 1999.
91. Zygun D. Hypothermia in severe traumatic brain injury: Questions remain. Journal of Critical Care. 2007 Sep;22(3):235-6.
92. Zygun D, Valadka A, Okonkwo D, Fourwinds S, Clifton G. Differential effect of hypothermia by operative status in severe traumatic brain injury: Can it be explained by differences of cerebral and systemic physiology? Critical Care Medicine. 2010;38:A91.
