# Supplementary figures and images for: A systematic review of therapeutic hypothermia for adult patients following traumatic brain injury
Source: Crit Care. 2014 Apr 17;18(2):R75. doi: 10.1186/cc13835 (PMC4056614; doi:10.1186/cc13835)

## Appendix 2. PRISMA DIAGRAM

### Flow Diagram: Study Selection Process

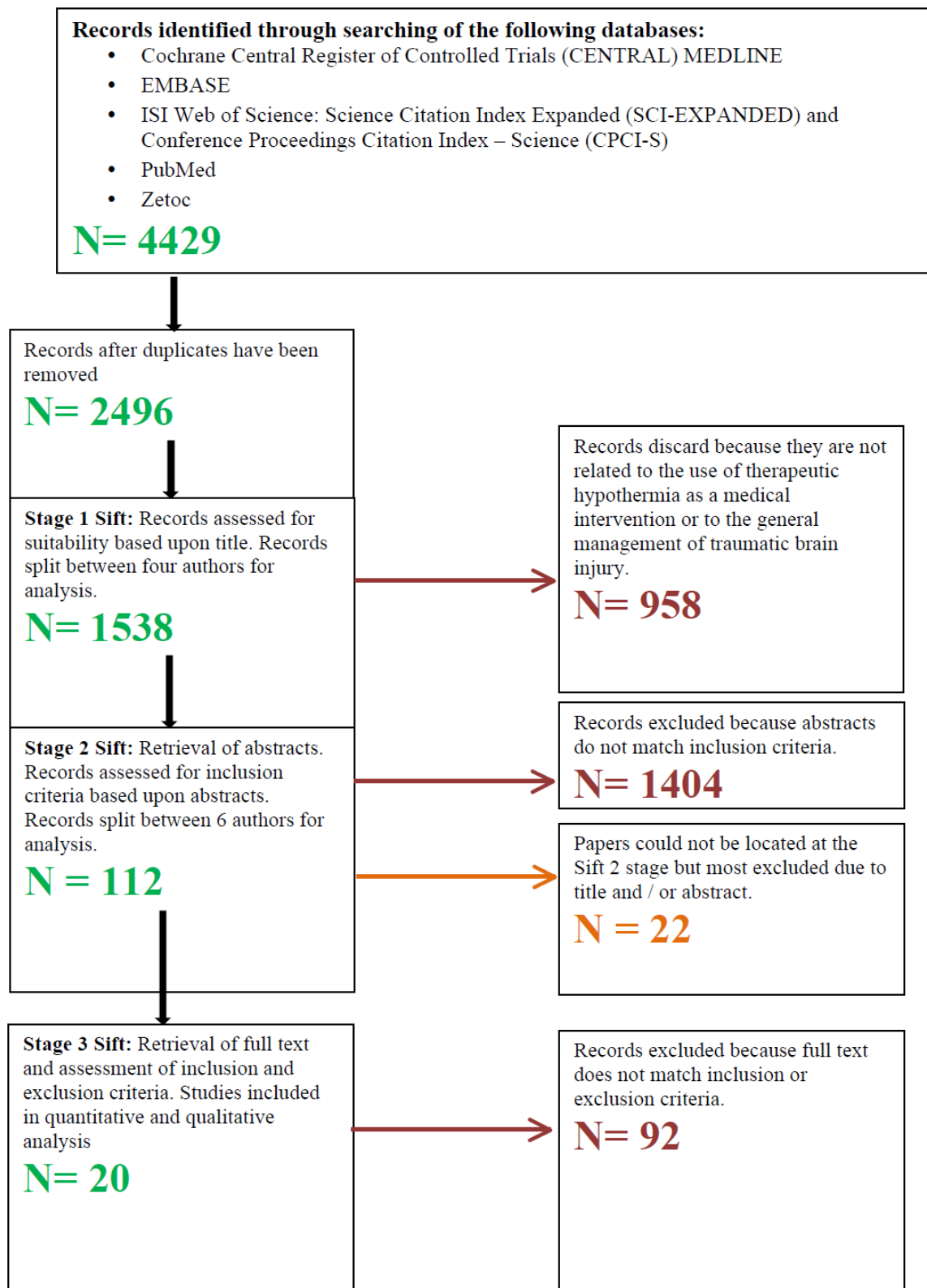

Supplement: Additional file 3 — PRISMA flow diagram showing the systematic review process. [file cc13835-S3.pdf]

**Appendix 3**. Domain-based assessment of the risk of bias


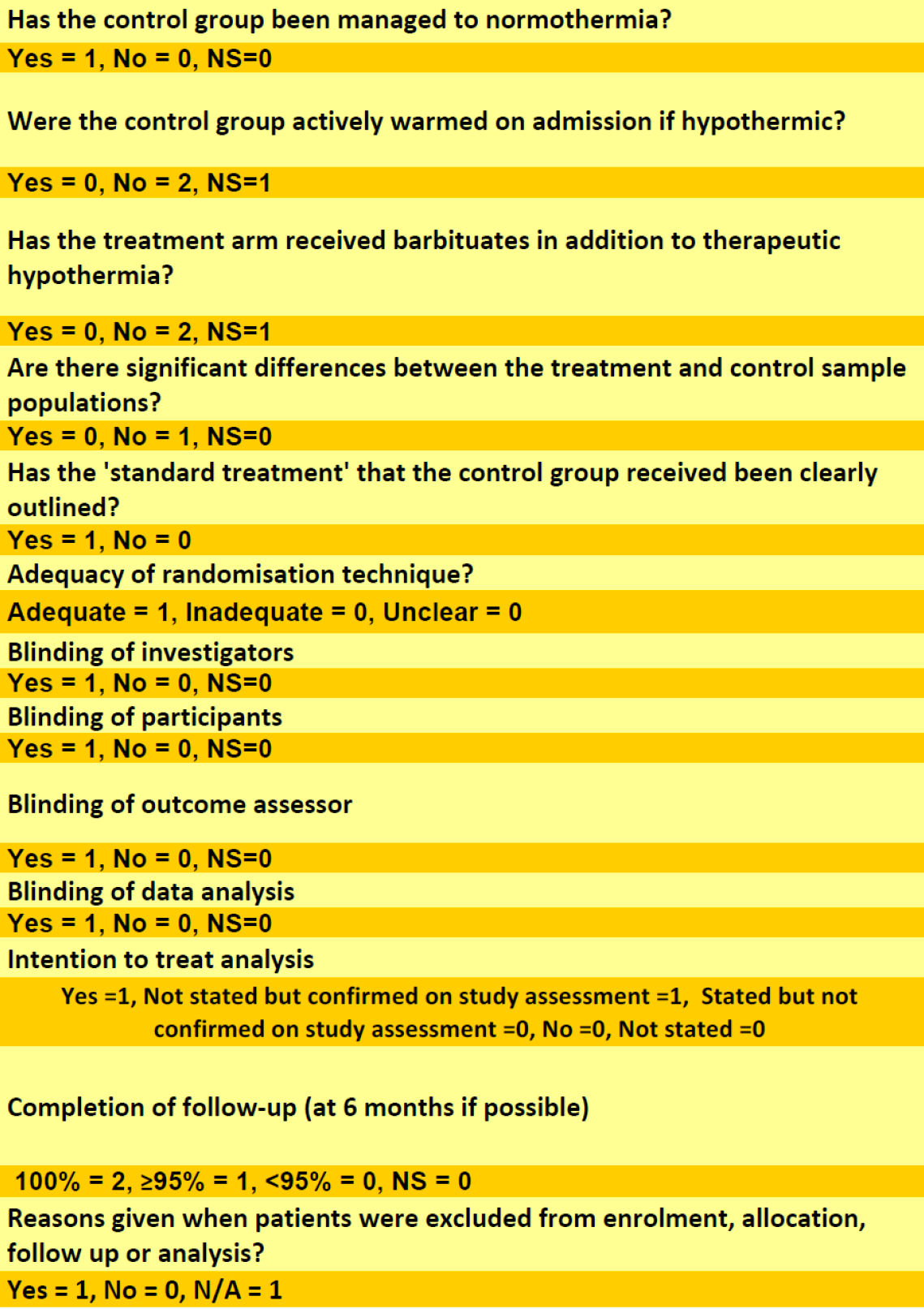


NS= not stated

Supplement: Additional file 4 — Domain-based assessment of risk of bias. [file cc13835-S4.doc]
