# Supplementary material for: A systematic review of therapeutic hypothermia for adult patients following traumatic brain injury
Source: Crit Care. 2014 Apr 17;18(2):R75. doi: 10.1186/cc13835 (PMC4056614; doi:10.1186/cc13835)
Supplement: Additional file 5 — Domain-based assessment of risk of bias. [file cc13835-S5.pdf]

	Clifton 1993	Clifton 2011	Clifton 2001	Gal 2002	Hashiguchi 2003	Jiang 2000	Lee 2010	Liu 2006	Marion 1997	Smrcka 2005	Zhao 2011	Zhi 2003	Maekawa 2009	Guo 2004	Guo 2004	Guo 2004	Guo 2004	Guo 2004	Guo 2004
**Has the control group been managed to normothermia?**	Yes	Yes	Yes	Yes	Yes	Yes	Yes	Yes	Yes	Yes	Yes	Yes	Yes	Yes	Yes	Yes	Yes	Yes	Yes
**Yes = 1, No = 0, NS=0**	1	1	1	1	1	1	1	1	1	1	1	1	1	1	1	1	1	1	1
**Were the control group actively warmed on admission if hypothermic?**	NS	No	NS	No but cooled to 36.5-37.5 if necessary	No (before randomisation 5 patients were given mild hypothermia (34C) as their ICP could not be maintained below 20mmHg using conventional therapy)	NS	NS	NS	No (but patients with rectal temp below 37°C on admission were passively re-warmed over 12 hours)	NS	NS	Yes	NS	No	NS	NS	NS	NS	NS
**Yes = 0, No = 2, NS=1**	1	2	1	2	2	1	1	1	2	1	1	0	1	2	1	1	1	1	1
**Has the treatment arm received barbituates in addition to therapeutic hypothermia?**	No	NS	Barbiturate coma was induced in patients whose intracranial pressure remained high.	No	Yes	No	NS	NS	NS	NS	NS	NS	NS	No	Yes	Yes	Yes	No	No
**Yes = 0, No = 2, NS=1**	2	1	0	2	0	2	1	1	1	1	1	1	1	2	0	0	0	2	2
**Are there significant differences between the treatment and control sample populations?**	No	Yes	No	No	Yes	No	No	No	No	No	Yes	Yes	NS	No	No	No	No	No	NS
**Yes = 0, No = 1, NS=0**	1	0	1	1	0	1	1	1	1	1	0	0	0	1	1	1	1	1	0
**Has the 'standard treatment' that the control group received been clearly outlined?**	Yes	Yes	Yes	Yes	Yes	No	Yes	No	Yes	No	Yes	Yes	No	Yes	Yes	No	Yes	No	Yes
**Yes = 1, No = 0**	1	1	1	1	1	0	1	0	1	0	1	1	0	1	1	0	1	0	1
**Adequacy of allocation concealment?**	Unclear	Adequate	Unclear	Inadequate	Unclear	Unclear	Unclear	Unclear	Adequate	Unclear	Unclear	Unclear	Unclear	Unclear	Adequate	Unclear	Unclear	Unclear	Unclear
**Adequate = 1, Inadequate &Unclear = 0**	0	1	0	0	0	0	0	0	1	0	0	0	0	0	1	0	0	0	0
**Blinding of investigators**	NS	No	NS	NS	NS	NS	NS	NS	NS	NS	NS	NS	NS	No	No	NS	No	NS	NS
**Yes = 1, No = 0, NS=0**	0	0	0	0	0	0	0	0	0	0	0	0	0	0	0	0	0	0	0
**Blinding of participants**	NS	No	NS	NS	NS	NS	NS	NS	NS	NS	NS	NS	NS	No	NS	NS	Yes	NS	NS
**Yes = 1, No = 0, NS=0**	0	0	0	0	0	0	0	0	0	0	0	0	0	0	0	0	0	0	0
**Blinding of outcome assesor**	GOS was assessed by a neuropsychologist who was blinded to treatment arm.	Yes	Yes	NS	NS	Yes	NS	NS	NS	NS	NS	NS	NS	NS	Yes	NS	NS	NS	NS
**Yes = 1, No = 0, NS=0**	1	1	1	0	0	1	0	0	0	0	0	0	0	0	1	0	0	0	0
**Blinding of data analysis**	NS	NS	No	NS	NS	NS	NS	NS	NS	NS	NS	NS	NS	NS	NS	NS	NS	NS	NS
**Yes = 1, No = 0, NS=0**	0	0	0	0	0	0	0	0	0	0	0	0	0	0	0	0	0	0	0
**Intention to treat analysis**	NS	Yes	Stated but not confirmed	NS	NS but confirmed	NS	NS	NS	NS but confirmed	NS but confirmed	NS	NS but confirmed	NS	NS but confirmed	NS but confirmed	NS but confirmed	NS but confirmed	NS	NS but confirmed
**Yes =1, Not stated but confirmed on study assessment =1, Stated but not confirmed on study assessment =0, No =0, Not stated =0**	0	1	0	0	1	0	0	0	1	1	0	1	0	1	1	1	1	0	1
**Completion of follow-up (at 6 months if poss)**	98%	100% in 3 months 92% at 6 months	7 (1.8%) lost to follow-up, data on age or GCS missing/inaccurate for 17 (4.3%) patients => 24 (6.1%) pnts excluded.	100%	100%	100%	100%	100%	100% at 6 months , 98.8% at 12 months	100%	100% at 3 months	100%	90%	100%	100% at 3 months	100%	100%	NS	89%
**100% = 2, ≥95% = 1, <95% = 0, NS = 0**	1	2	0	2	2	2	2	2	2	2	2	2	0	2	2	2	2	0	0
**Reasons given when pateints were excluded from enrollment, allocation, follow up or analysis?**	No	Yes	Yes	No	Yes	No	Yes	No	Yes	Yes	Yes	No	No	No	Yes	Yes	Yes	No	Yes
**Yes = 1, No = 0, N/A = 1**	0	1	1	0	1	0	1	0	1	1	1	0	0	0	1	1	1	0	1
**TOTAL (Number of Domaines)**	8	11	6	9	8	8	8	7	11	8	7	6	3	10	10	7	8	5	7
**MAXIMUM (Number of Domaines)**	15	15	15	15	15	15	15	15	15	15	15	15	15	15	15	15	15	15	15

NS = Not stated
