# Supplementary material for: A systematic review of therapeutic hypothermia for adult patients following traumatic brain injury
Source: Crit Care. 2014 Apr 17;18(2):R75. doi: 10.1186/cc13835 (PMC4056614; doi:10.1186/cc13835)
Supplement: Additional file 6 — Characteristics of all selected trials. [file cc13835-S6.doc]

Characteristics of the 20 Included Trials.
