# Supplementary material for: A systematic review of therapeutic hypothermia for adult patients following traumatic brain injury
Source: Crit Care. 2014 Apr 17;18(2):R75. doi: 10.1186/cc13835 (PMC4056614; doi:10.1186/cc13835)
Supplement: Additional file 7 — Prospero protocol for this systematic review. [file cc13835-S7.pdf]

PROSPERO
International prospective register of systematic reviews

NHS
National Institute for Health Research

Systematic review for the treatment of traumatic brain injury using therapeutic hypothermia

Clair Clark, Judith Hayton, Imogen Murray, Samantha Crossley, Jenny Reid, Rachel McLatchie, Peter Andrews, Margaret MacDougall

Citation

Clair Clark, Judith Hayton, Imogen Murray, Samantha Crossley, Jenny Reid, Rachel McLatchie, Peter Andrews, Margaret MacDougall. Systematic review for the treatment of traumatic brain injury using therapeutic hypothermia. PROSPERO 2012:CRD42012002449 Available from
http://www.crd.york.ac.uk/PROSPERO/display_record.asp?ID=CRD42012002449

Review question(s)

The main objective of this review is to assess the effects of the application of therapeutic hypothermia when administered to adult patients who have been admitted to hospital following traumatic brain injury.

Searches

Searches will not be restricted by date, language or publication status (other than those restrictions imposed by the databases themselves). Where necessary papers will be translated. We will search the following electronic databases:

- Cochrane Central Register of Controlled Trials (CENTRAL)

- MEDLINE

- EMBASE

- ISI Web of Science: Science Citation Index Expanded (SCI-EXPANDED) and Conference Proceedings Citation Index – Science (CPCI-S)

- PubMed

- Zetoc

Search strategy can be found in appendix II of the protocol.

Types of study to be included

Inclusion criteria:

• Trials must be randomised control trials.

Exclusion criteria:

• Trials in which patients have not been randomised to each treatment arm and/or where there is no control group managed to normothermia.

Condition or domain being studied

The domain being studied is the application of therapeutic hypothermia for the treatment of traumatic brain injury. We define traumatic brain injury as being any acute closed head injury sustained following head trauma.

We define therapeutic hypothermia as any intervention carried out with the intention of reducing core body temperature to below the physiological norm (36.0 degrees Celsius). Unfavourable outcomes at the end of the follow up period will include death, persistent vegetative state or severe disability as defined by the Glasgow Outcome Scale or equivalent scoring scale (Ranchos Los Amigos Scale).

Participants/ population

Inclusion criteria:

• Patients must be adults. We define this as being the legal age for consent in the country in which the trial was conducted.

• Patients enrolled must have closed head injuries.

Exclusion criteria:

• The trial has been performed entirely in neonates or children, whom we define as being below the legal age for

consent.

• Trials containing patients with open head injuries such as gun shot wounds will be excluded.

Intervention(s), exposure(s)

The intervention under review is therapeutic hypothermia. All methods of inducing hypothermia will be included in this review; the intervention used will be identified in the data extraction form. Such interventions will include, but will not be limited to, cooling blankets, gastric lavage, intravenous cooling techniques and application of cold packs. Use of barbituates is not a reason for exclusion but their inclusion in treatment will also be documented in the data extraction form.

Comparator(s)/ control

Inclusion criteria:

- Trials must have a control group that is treated to normothermia, which is defined as standard body temperature.

Exclusion criteria:

- Control group is not managed to normothermia.

Outcome(s)

Primary outcomes

Our primary outcome is to assess the effects of the application of therapeutic hypothermia when administered to adult patients who have been admitted to hospital following traumatic brain injury.

Secondary outcomes

Our secondary outcomes are to investigate the following hypothesis:

a. Therapeutic hypothermia reduces the risk of a poor outcome following adult traumatic brain injury.

b. Duration of cooling lasting greater than 48 hours confers improved outcome compared with cooling of less than this duration.

c. Re-warming patients at a speed greater than 1 degree Celsius every four hours increases the risk of poor outcome.

d. Patients who have undergone only modest cooling (35-36 degrees Celsius) experience greater poor outcomes compared with patients cooled to below 35 degrees Celsius.

e. Increased length of time between the onset of injury and induction of cooling increases the risk of poor outcome.

Data extraction, (selection and coding)

The primary output of the searches will be divided amongst four authors who will discard studies that are not related to the use of therapeutic hypothermia as a medical intervention or to the general management of traumatic brain injury, recording reasons for their removal from this analysis. Six authors will then retrieve abstracts and full text of the remaining papers. Each study will be assessed independently by two authors for inclusion criteria. Abstracts that match exclusion criteria will be disregarded. Where authors are uncertain and discrepancies remain about studies, a majority decision between the six authors and a clinical specialist (Professor Peter Andrews) will determine if the study meets inclusion criteria. The titles, abstracts and content of non-English language papers will be translated. The number of papers at each of the stages of searching will be documented also, in order that it is clear to readers how the final set of papers was reached. A PRISMA flow diagram will be used to guide this process.

Risk of bias (quality) assessment

The following proforma will be used to asses the risk of bias. This will be included in the data extraction form.

a. Allocation Concealment:

Document randomisation technique used plus:

i. Adequate - randomisation method described that would not allow investigator or

participant to know or influence intervention group before eligible participant entered in the study.

ii. Unclear - Randomisation stated but no information on method used is available.

iii. Inadequate - Method of randomisation used such as alternate medical record numbers or unsealed envelopes; any information in the study that indicated that investigators or participants could influence the intervention group.

b. Blinding:

Blinding of investigators: Yes/No/not stated

Blinding of participants: Yes/No/not stated

Blinding of outcome assessor: Yes/No/not stated

Blinding of data analysis: Yes/No/not stated

The above are not considered blinded if the treatment group can be identified in > 20% of

participants because of the side effects of treatment.

c. Intention-to-treat Analysis:

Yes - specifically reported by the authors that intention-to-treat analysis was undertaken and this was confirmed on study assessment.

Yes - not stated, but confirmed on study assessment.

No - not reported and lack of intention-to-treat analysis confirmed on study assessment.

(Patients who were randomised were not included in the analysis because they did not receive the study intervention, they withdrew from the study, or were not included because of protocol violation).

No - stated but not confirmed upon study assessment.

Not stated.

d. Completeness of follow-up

Percentage of patients excluded or lost to follow-up.

Strategy for data synthesis
Data used will be aggregate and both quantitative and descriptive data synthesis is planned.

For temperature data the difference in means will be calculated with 95% confidence intervals. If there are sufficient good quality trials for a meta-analysis a weighted mean difference will be calculated. In this case a clinically significant size of effect of 5% has been decided upon.

Statistical heterogeneity will be calculated using the chi-squared test and I-squared index. Depending on the outcome of this test and whether there is true heterogeneity between the results of studies, pooled relative risk and 95% confidence intervals for all-cause mortality and improved neurological outcome will be calculated using either a random-effects model or fixed effect model. Subsequent models may be applied should the data be suitable for meta-analysis.

It is likely to be appropriate to conduct sensitivity analyses of some aspects of therapeutic hypothermia, in relation to all-cause mortality for example, but it is difficult to pre-specify these precisely. Factors which may be relevant include target temperature, cooling rate, duration and rate of rewarming.

Analysis of subgroups or subsets
None planned at present.

Dissemination plans
Review is for publication with the European Society of Intensive Care Medicine

Contact details for further information
Clair Clark

University of Edinburgh Medical School

47 Little France Crescent

Edinburgh

Midlothian EH16 4TJ

s0901852@sms.ed.ac.uk

Organisational affiliation of the review
University of Edinburgh Medical School & Lothian Hospitals University Division. Also The European Society of Intensive Care Medicine

http://www.ed.ac.uk/schools-departments/medicine-vet-medicine

Review team
Miss Clair Clark, Medical Student
Miss Judith Hayton, Medical Student
Mrs Imogen Murray, Medical Student
Miss Samantha Crossley, Medical Student
Miss Jenny Reid, Medical Student
Miss Rachel McLatchie, Medical Student
Professor Peter Andrews, Professor in Anesthetics & Intensive Care
Dr Margaret MacDougall, Medical Statistician University of Edinburgh

Anticipated or actual start date
01 August 2011

Anticipated completion date
22 October 2012

Funding sources/sponsors

None

Conflicts of interest
None known

Other registration details
Review title is listed with the European Society of Intensive Care Medicine

Language
English

Country
Scotland

Subject index terms status
Subject indexing assigned by CRD

Subject index terms
Brain Injuries; Humans; Hypothermia, Induced

Reference and/or URL for protocol
http://www.crd.york.ac.uk/PROSPEROFILES/2449_PROTOCOL_20120705.pdf

Date of registration in PROSPERO
21 June 2012

Date of publication of this revision
09 August 2012

Stage of review at time of this submission	Started	Completed
Preliminary searches	No	Yes
Piloting of the study selection process	No	Yes
Formal screening of search results against eligibility criteria	No	Yes
Data extraction	Yes	No
Risk of bias (quality) assessment	No	No
Data analysis	No	No
Prospective meta-analysis	No	No

PROSPERO
This information has been provided by the named contact for this review. CRD has accepted this information in good faith and registered the review in PROSPERO. CRD bears no responsibility or liability for the content of this registration record, any associated files or external websites.
