# Supplementary material for: A systematic review of therapeutic hypothermia for adult patients following traumatic brain injury
Source: Crit Care. 2014 Apr 17;18(2):R75. doi: 10.1186/cc13835 (PMC4056614; doi:10.1186/cc13835)
Supplement: Additional file 8 — Differences between this systematic review and the most recent Cochrane review. [file cc13835-S8.pdf]

5. Differences between this systematic review and the most recent Cochrane review

Papers in Cochrane Review: Differences between selections of studies for analysis.

Paper	Decision	Reason
Adelson 2005 HYPO1	Exclusion	Paediatric patients
Adelson 2005 HYPO2	Exclusion	Paediatric patients
Aibiki 2000	Exclusion	Paediatric patients
Biswas 2002	Exclusion	Paediatric patients
Clifton 1992	Cannot access – author unable to provide copy of paper	
Clifton 1993	Inclusion	Met inclusion criteria
Clifton 2001	Inclusion	Met inclusion criteria
Harris 2009	Exclusion	Not measuring GOS
Hashiguchi 2003	Inclusion	Met inclusion criteria
Hirayama 1994	Inclusion	Met inclusion criteria
Hutchison 2008	Exclusion	Paediatric patients
Ishikura 1998	Exclusion	Symposium presentation
Jiang 2000	Inclusion	Met inclusion criteria
Marion 1997	Inclusion	Met inclusion criteria
Meissner 2003a	Exclusion	Not measuring GOS, thyroid function only
Meissner 2003b	Exclusion	Not measuring GOS, thyroid function only
Qiu 2007	Inclusion	Met inclusion criteria
Shiozaki 1993	Inclusion	Met inclusion criteria
Shiozaki 1999	Inclusion	Met inclusion criteria
Shiozaki 2001	Exclusion	Paediatric patients
Smrcka 2005	Included	Met inclusion criteria
Yan 2001	Exclusion	Not measuring GOS
Zhang 2000	Cannot access paper	
